# Side‐Chain‐Induced Interlayer Slipping in Metalloporphyrin COFs Enables Microenvironment and Spin‐State Regulation for Photocatalytic CO_2_ Reduction

**DOI:** 10.1002/adma.74747

**Published:** 2026-08-22

**Authors:** Jie He, Qingxuan Chen, Minxian Zhang, Wenhao Zhao, Aoni Xu, Jinqiang Zhang, Hongqi Sun, Shaobin Wang, Xiaoguang Duan

**Affiliations:** ^1^ School of Chemical Engineering Adelaide University Adelaide South Australia Australia; ^2^ School of Chemical and Biomolecular Engineering and ARC Centre of Excellence for Green Electrochemical Transformation of Carbon Dioxide The University of Sydney Sydney New South Wales Australia; ^3^ School of Molecular Sciences The University of Western Australia Perth Western Australia Australia

**Keywords:** high‐spin effect, interlayer slipping, photocatalytic CO_2_ reduction, porphyrin‐COFs, structural regulation

## Abstract

Photocatalytic CO_2_ reduction (CO_2_RR) involves a cascade of intrinsically coupled processes, rendering the independent optimization of catalytic kinetics and thermodynamics challenging. In this study, we introduce a structural regulation strategy via steric‐driven interlayer slipping engineering of metalloporphyrin‐based covalent organic frameworks (COFs) to decouple and simultaneously optimize the catalytic microenvironment and the spin state of the active metal center. Through regulating the length of alkoxy side‐chains, conventional AA stacking is transformed into a moderately serrated slipped configuration (AA*). Kinetically, the resulting slipped nanochannels enhance local hydrophobicity and spatial confinement, thereby greatly enriching in‐channel CO_2_ concentration and suppressing hydrogen evolution. Thermodynamically and electronically, this interlayer slipping reconstructs the ligand field of catalytic Co sites and maximizes vertical π–d exchange interactions. As a result, this triggers a collective spin transition from isolated low‐spin (*S* = 1/2) monomers to a high‐spin (*S* = 3/2) state, which significantly prolongs charge carrier lifetimes and optimizes the adsorption and activation of the *COOH intermediate. Consequently, the optimized CoP‐COFs deliver a record CO production rate of 71.4 mmol g^−1^ h^−1^ with 90% selectivity among porphyrin‐based COF photocatalysts. This work establishes stacking engineering as a versatile strategy for decoupling entangled reaction steps to enable efficient solar fuel production.

## Graphical abstract

Alkoxy chain length drives a continuous transition from eclipsed AA to a serrated, slipped AA* configuration over Co‐porphyrin COFs. A moderate slip both contracts the pore channel to enrich CO_2_ and suppress hydrogen evolution, and enhances collective high‐spin of Co sites, which delivers a record CO production rate with high selectivity.

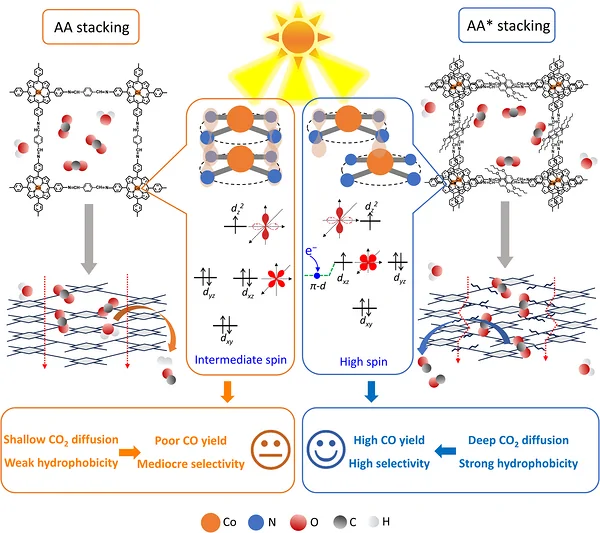

## 1 Introduction

Photocatalytic CO_2_ reduction (CO_2_RR) represents a long‐sought route to convert solar energy into chemical fuels and close the anthropogenic carbon cycle [1, 2]. Despite substantial advances in catalyst design, achieving simultaneously high activity, and selectivity remains fundamentally challenging because CO_2_RR is governed by multiple strongly coupled elementary processes. These include CO_2_ diffusion and adsorption, suppression of the competing hydrogen evolution reaction (HER), stabilization, and transformation of diverse key intermediates (e.g., *COOH and *CO), as well as efficient separation, transport, and use of photogenerated charge carriers [3, 4]. Critically, these catalytic subprocesses impose distinct and often conflicting structural requirements. For instance, enhancing CO_2_ adsorption often necessitates polar or coordinatively active sites, whereas suppressing HER favors hydrophobic environments. Likewise, strong binding of intermediates can promote activation but hinder product desorption [5, 6, 7, 8]. In conventional catalyst design, these multiple reaction parameters are predominantly governed by a limited set of descriptors, such as metal species, coordination environment, and interfacial chemistry (e.g., hydrogen‐bonding networks) [9, 10, 11]. Consequently, the key processes in CO_2_RR are intrinsically coupled, as optimizing one reaction step often compromises another. Traditional active‐site‐centered engineering strategies lack the sufficient tuning dimensions needed to independently address the multistep complexity of CO_2_RR. This bottleneck highlights an urgent need for orthogonal structural regulation strategies that can introduce additional, independently tunable variables into the catalyst architecture.

Covalent organic frameworks (COFs) offer a promising platform to address this challenge owing to their highly ordered architecture, tunable porosity, and modular design [12, 13, 14]. In particular, metalloporphyrin‐based two‐dimensional (2D) COFs enable the precise incorporation of atomically defined metal centers into extended π‐conjugated networks, thereby integrating catalytic active sites with long‐range charge transport pathways [15, 16]. In such systems, tunable functionalized monomers play a critical role in determining charge transport, electronic coupling, and pore affinity [17, 18]. For example, alkoxy‐chain functionalization has been used to tailor pore hydrophilicity–hydrophobicity and enhance both multiphase mass transfer and catalytic performance in CO_2_RR, oxygen reduction reaction (ORR), or HER [19, 20, 21, 22]. Nevertheless, most studies implicitly assume ideal eclipsed AA stacking when introducing side‐chains. By attributing the enhanced catalytic performance to synergistic electronic and hydrophobic effects of long alkoxy substituents, they often overlook a critical reality: the interlayer stacking is highly susceptible to steric perturbations imposed by these functional groups.

Actually, interlayer slipping driven by steric effects and energy minimization is ubiquitous [23]. To alleviate steric congestion from overlapping side‐chains and maintain structural stability, COF layers tend to undergo lateral reorganization in their interlayer π–π stacking [24, 25], leading to slipped AA (AA*) or even AB stacking arrangements [26, 27]. Steric tuning has been demonstrated as an effective strategy to control AA, AB, and ABC stacking modes in 2D COFs [28]. More recently, pendant alkyl/alkoxy side‐chain length was shown to strongly influence both the crystallinity and stacking geometry of imine‐linked 2D COFs, confirming that side‐chain interactions modulate the interlayer arrangement [27]. Theoretical studies have further revealed that fully eclipsed AA stacking is not always the lowest‐energy configuration, because lateral displacement between adjacent COF layers can reduce interlayer Pauli repulsion and steric congestion, thereby generating serrated or slipped AA‐type stacking motifs [29]. Interlayer displacement in COFs can also be dynamic and sensitive to local host–guest interactions; for example, Zhao's group reported that 2D COFs can undergo interlayer shifting in response to solvent or gas adsorption [30, 31]. In contrast, the interlayer slipping driven by energy minimization through covalently anchored functional groups is thermodynamically stable and can effectively stabilize a specific stacking configuration within the COF. Such slip‐induced stacking shifts can alter the π–π interactions between adjacent layers, thereby regulating the electronic structure of metal single‐atom centers coordinated within the COF [32]. Therefore, side‐chain‐induced modulation of interlayer stacking geometry may serve as an orthogonal structural regulator that simultaneously optimizes the catalytic microenvironment and the electronic structure. However, whether this often‐overlooked structural variable can be deliberately engineered to decouple the intrinsically coupled processes in CO_2_RR remains ambiguous.

In this study, we demonstrate that controlled interlayer slipping in metalloporphyrin COFs transcends merely a structural deviation, providing instead as an orthogonal control dimension to decouple and optimize complex photocatalytic behaviors. Using Co‐porphyrin COFs (CoP‐COFs) as a model material, we employed steric side‐chain engineering to drive a controlled transition from ideal AA stacking to a serrated, slipped configuration (AA* stacking). This structural modulation simultaneously, yet independently, reconstructs the nanochannel environment and interlayer electronic interactions. The slipped nanochannels create a confined and highly hydrophobic microenvironment that enhances CO_2_ enrichment while suppressing hydrogen evolution. Furthermore, such interlayer sliding maximizes ferromagnetic coupling along the vertical Co─Co alignment through optimized π–d exchange interactions. This cooperative magnetic interaction leads to a collective high‐spin state (*S* = 3/2) that prolongs photogenerated electron lifetimes and accelerates the activation of key CO_2_RR intermediates. By systematically tuning alkoxy side‐chain lengths, this work establishes stacking engineering as a multiscale paradigm, seamlessly unifying spin‐state physics with microenvironment design to overcome the intrinsic limitations of multistep catalysis.

## 2 Results and Discussion

### 2.1 Structural Characterizations of CoP‐COFs With Different Side‐Chain Lengths

Imine‐linked covalent organic frameworks (CoP‐COFs) with systematically varied alkoxy side‐chain lengths were synthesized via a conventional solvothermal Schiff‐base condensation between Co‐coordinated porphyrin monomers (Co‐Por) and dialdehyde linkers (Figure S1). By tuning the alkoxy substituents, a series of CoP‐COFs with increased carbon‐chain lengths of C_0_, C_1_, C_5_, and C_12_ were obtained, denoted as CoP‐TTA‐COF (C_0_), CoP‐DMTA‐COF (C_1_), CoP‐BpTA‐COF (C_5_), and CoP‐BdTA‐COF (C_12_), respectively. The structures of the porphyrin monomer and porphyrin COFs were confirmed by solid‐state ^13^C nuclear magnetic resonance (NMR) spectroscopy (Figures S2–S6). All samples exhibit major characteristic peaks at 143.6, 134.3, and 115.2 ppm, corresponding to the carbon framework of the porphyrin macrocycle. Notably, a distinct resonance at 154.2 ppm is observed in all COF samples and is attributed to the imine carbon, clearly distinguishing the COFs from the monomer Co‐Por‐NH_2_ precursor. For CoP‐DMTA‐COF, CoP‐BpTA‐COF, and CoP‐BdTA‐COF, an additional peak at 69.2 ppm is observed, corresponding to the α‐carbon of the alkoxy side‐chain. Moreover, CoP‐BpTA‐COF and CoP‐BdTA‐COF display further signals at 29.3 and 14.2 ppm, which can be attributed to the additional carbons of the extended alkoxy chains, respectively, confirming the successful incorporation of longer side‐chains into the COF scaffold.

Fourier transform infrared (FTIR) spectra show that all CoP‐COFs and Co‐Por‐NH_2_ monomer exhibit a characteristic Co─N stretching vibration at 530 cm^−1^ (Figure S7). In contrast, the control sample of H_2_N_4_‐BpTA‐COF, synthesized without embedded Co, shows no such feature, confirming successful metal incorporation into the COF framework. In addition, a distinct ─C═N─ vibration band at 1620 cm^−1^ is observed in all CoP‐COF samples but absent in Co‐Por‐NH_2_, demonstrating the successful formation of COF structures. Notably, CoP‐TTA‐COF exhibits a red‐shifted ─C═N─ band relative to the COFs bearing alkoxy side‐chains, indicating that alkoxy substitution slightly perturbs the local chemical environment of the imine linkage; further increases in alkoxy‐chain length, however, produces no additional pronounced shift. The initial shift from CoP‐TTA‐COF to alkoxy‐chain‐containing COFs can be ascribed to the weak electron‐donating inductive effect and steric influence of introduced alkoxy side‐chains [27, 33]. However, the electron‐donating inductive effect of alkoxy groups is inherently short‐ranged and decays rapidly with increasing σ‐bond distance, which does not substantially alter the local vibrational environment of the imine linkage as side‐chain length increases [34, 35]. The intensities of v_s‐CH_2_ (2852 cm^−1^) and v_as‐CH_3_ (2954 cm^−1^) stretching vibrations rise with chain length, further confirming successful modification with the alkoxy side‐chains.

As illustrated in Figure 1a, varying the alkoxy side‐chain length provides an effective means to modulate the slip degree in AA‐stacked CoP‐COFs, driven by the steric hindrance effect of functionalized long chains. Based on this, PXRD patterns and reproduced structures of these four CoP‐COFs are collected and displayed in Figure 1b–i. All CoP‐COFs exhibit main diffraction peaks at approximately 3.5°, 4.1°, 7.0°, and 8.4°, which can be indexed to the (100), (010), (200), and (020) planes, respectively. These peaks are confirmed to be distinct from any residual monomers (Figures S8 and S9). Simulated PXRD patterns were compared with experimental data using Pawley refinement. For CoP‐TTA‐COF (C_0_) and CoP‐DMTA‐COF (C_1_), the simulated patterns based on eclipsed AA stacking show excellent agreement with the experimental profiles (orange curves in Figure 1b,d), suggesting a predominantly face‐to‐face stacking mode in these two samples.

**FIGURE 1:**
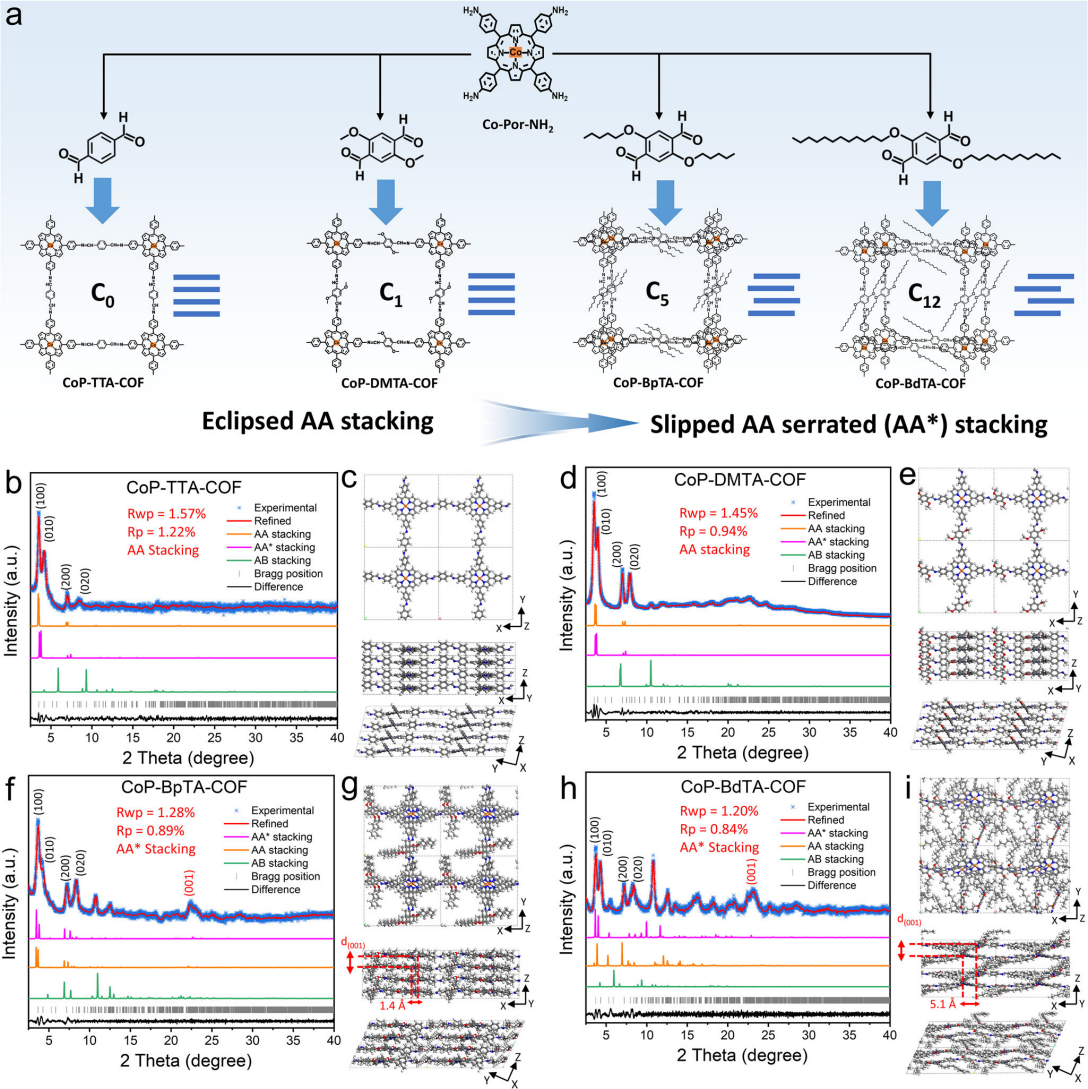
Synthesis and structural characterization of CoP‐COFs. (a) Schematic illustration of synthesis of CoP‐COFs with different side‐chain lengths. PXRD patterns of (b) CoP‐TTA‐COF, (d) CoP‐DMTA‐COF, (f) CoP‐BpTA‐COF, and (h) CoP‐BdTA‐COF with experimental curves, Pawley refinement profiles, corresponding difference plots, simulated patterns for AA stacking, AA* stacking and AB stacking mode, and Bragg reflection positions. Reconstructed four‐layer crystal structures of (c) CoP‐TTA‐COF, (e) CoP‐DMTA‐COF, (g) CoP‐BpTA‐COF, and (i) CoP‐BdTA‐COF (grey, C; white, H; blue, N; red, O; orange, Co).

Notably, when an orthorhombic lattice (*α* = *β* = *γ* = 90°) is assumed for CoP‐TTA‐COF, the simulated XRD pattern fails to reproduce the experimental diffraction features, particularly the reflections corresponding to (010) and (020) planes (Figure S10a,b). Typically, a single sharp Bragg diffraction peak associated with the (100) plane reflects high crystallinity and a highly symmetric unit cell. However, when the lattice is tilted and structural symmetry is reduced, variations in interplanar distances lead to peak splitting, giving rise to distinguishable (100) and (010) reflections [27, 36]. Upon releasing the constraint on the α angle during structural optimization, the simulated PXRD patterns become consistent with the experimental data, yielding an optimized α angle of 103°. These results indicate that these COFs do not adopt the strictly fourfold in‐plane symmetry with an idealized P4/m space group. Accordingly, subsequent structural optimizations were performed without constraining the α lattice angles, and the structures were best described as an inclined stacking of monoclinic frameworks. Under these conditions, the AA‐stacked CoP‐TTA‐COF adopts a *P*1 lattice with *α* = 103°, β = *γ* = 90°, *a* = 25.7 Å, *b* = 25.8 Å, and *c* = 4.4 Å, while CoP‐DMTA‐COF converges to a *P*1 lattice with *α* = 105°, *β* = *γ* = 90°, *a* = 25.8 Å, *b* = 25.7 Å, and *c* = 4.3 Å (Figure 1c,e). In addition, neither simulation based on AB stacking nor slipping AA serrated configurations (AA*) stacking models reproduces the experimental XRD patterns in terms of peak position or intensity for both CoP‐TTA‐COF and CoP‐DMTA‐COF (Figures S10c and S11), confirming that the two COFs follow the eclipsed AA stacking mode along the *z* axis.

For CoP‐BpTA‐COF and CoP‐BdTA‐COF bearing longer side‐chains (≥C_5_), a face‐to‐face eclipsed AA stacking model fails to reproduce the experimental results in terms of peak position or intensity (orange curves in Figure 1f,h). High‐resolution PXRD measurements were further conducted for structural refinement, using a smaller step size and a slower scan rate. As shown in Figure S12, the resulting PXRD patterns show improved signal quality compared with the standard PXRD data, enabling clearer identification of the structural features. Comparison of the high‐resolution PXRD patterns of the four COFs reveals that CoP‐BpTA‐COF and CoP‐BdTA‐COF exhibit markedly decreased diffraction intensities relative to CoP‐TTA‐COF and CoP‐DMTA‐COF, along with slight shifts of the main (100) planes. These changes can be attributed to the alkoxy‐chain‐induced modulation of interlayer interactions in the 2D imine‐linked COF frameworks, which promotes slipped stacking. The resulting stacking modulation, together with pore contraction and reduced d‐spacing, leads to an altered periodic pore arrangement, reduced crystallinity, and incomplete in‐plane overlap between adjacent layers [27, 37]. Consequently, the low‐angle PXRD reflections, particularly the (100) planes, show slight peak shifts and intensity variations. Meanwhile, a weak superlattice reflection emerges at around 5.5°, accompanied by the appearance of a sharp (001) reflection at about 23°, indicating the formation of a new short‐range periodicity along the *z* axis. This structural transition is attributed to the substantial steric hindrance imposed by the extended alkoxy side‐chains. To alleviate this steric strain, the framework deviates from ideal AA stacking via alternating interlayer offsets, ultimately forming a two‐layer supercell characterised by a serrated slipped configuration (denoted as AA*) [31, 38].

To quantitatively describe this structural transformation, PXRD simulations were performed by introducing controlled layer‐serrated slipping along the *x*‐axis on the basis of conventional AA stacking. The simulated patterns of AA* stacking (purple curves) with slipping distances of 1.4 and 5.1 Å show excellent agreement with the experimental data for CoP‐BpTA‐COF and CoP‐BdTA‐COF, respectively, (Figure 1f,h). In contrast, neither ideal AA stacking nor slipped configurations at other distances reproduces the experimental profiles satisfactorily (Figures S13–S15), underscoring the necessity of a well‐defined slipped structure. Density functional theory (DFT) calculations further corroborate this conclusion: the energetically favored configuration for CoP‐BpTA‐COF and CoP‐BdTA‐COF corresponds to a serrated slipped structure with an offset of 1.5 and 5.0 Å (Figure S16), whereas for CoP‐TTA‐COF and CoP‐DMTA‐COF, non‐slipped AA stacking (0 Å) is energetically more favorable than slipped AA* stacking (1.5 Å), consistent with the PXRD simulations. The stabilization of these structures is primarily driven by the reduction of interlayer Pauli repulsion [29]. These results collectively demonstrate that CoP‐BpTA‐COF and CoP‐BdTA‐COF adopt a serrated AA* stacking mode, with the degree of interlayer displacement increasing with side‐chain length (Figure 1g,i). The optimized structures can be described by a *P*1 lattice, with parameters of *α* = 114.8°, *β* = *γ* = 90°, *a* = 25.5 Å, *b* = 25.6 Å, *c* = 4.3 Å for CoP‐BpTA‐COF, and *α* = 114.9°, *β* = *γ* = 90°, *a* = 25.6 Å, *b* = 25.5 Å, *c* = 5.3 Å for CoP‐BdTA‐COF. To resolve the slipping difference more rigorously, Rietveld refinements of CoP‐BpTA‐COF and CoP‐BdTA‐COF were performed at 300 K and 0.1 MPa using a Bragg‐Brentano geometry. As shown in Figure S17, the two refinement approaches for CoP‐BpTA‐COF and CoP‐BdTA‐COF give highly consistent apparent slipping distances along the *x* axis (1.40 Å for CoP‐BpTA‐COF from both methods, and 5.1 and 4.8 Å for CoP‐BdTA‐COF from Pawley and Rietveld refinements, respectively). This agreement indicates that the proposed slipping trend is robust with respect to the refinement method and supports the assignment of moderate slipped stacking in CoP‐BpTA‐COF and more pronounced slipping in CoP‐BdTA‐COF.

Scanning electron microscope (SEM) images reveal that CoP‐TTA‐COF exhibits a smooth and well‐defined spherical morphology, while progressively longer side‐chains, C_1_ in CoP‐DMTA‐COF, C_5_ in CoP‐BpTA‐COF, and C_12_ in CoP‐BdTA‐COF, produce increasingly rough surfaces (Figure S18). This microscopic morphological evolution indicates that the alkoxy side‐chains disrupt interlayer structural regularity and π–π interactions, ultimately facilitating layer slipping and the formation of serrated stacking configurations. Notably, CoP‐BdTA‐COF undergoes partial structural collapse, indicating that the excessive steric bulk of the extended side‐chains drives interlayer displacement beyond the stability threshold of the COF framework. High‐angle annular dark‐field scanning transmission electron microscopy (HAADF‐STEM) results further support this structural evolution. As shown in Figure 2a–d, the overall morphologies are consistent with SEM observations. At higher magnification, well‐defined in‐plane lattice arrangements of the 2D porphyrin frameworks are clearly visible (Figure 2e–h). Notably, the lattice spacing in CoP‐BpTA‐COF (1.59 nm) and CoP‐BdTA‐COF (1.20 nm) is significantly reduced compared with the 1.95 and 1.91 nm spacing observed in CoP‐TTA‐COF and CoP‐DMTA‐COF, indicating a contraction of the in‐plane periodicity associated with interlayer slipping. Selected‐area electron diffraction (SAED) patterns from Fourier‐transformed images provide further insight into the structural changes. While CoP‐TTA‐COF and CoP‐DMTA‐COF exhibit well‐defined tetragonal diffractions, CoP‐BpTA‐COF and CoP‐BdTA‐COF display additional satellite diffraction spots, indicative of lattice distortion and superstructure formation. These observations confirm that long side‐chain‐induced interlayer slipping alters the π–π stacking registry, leading to lattice relaxation and modified structural symmetry within the COF frameworks. By contrast, HAADF‐STEM of monomer Co‐Por‐NH_2_ does not reveal lattice fringes or ordered periodic domains but instead shows isolated bright spots consistent with single‐atom dispersion, indicating the absence of long‐range crystalline π‐stacked packing (Figure S19).

**FIGURE 2:**
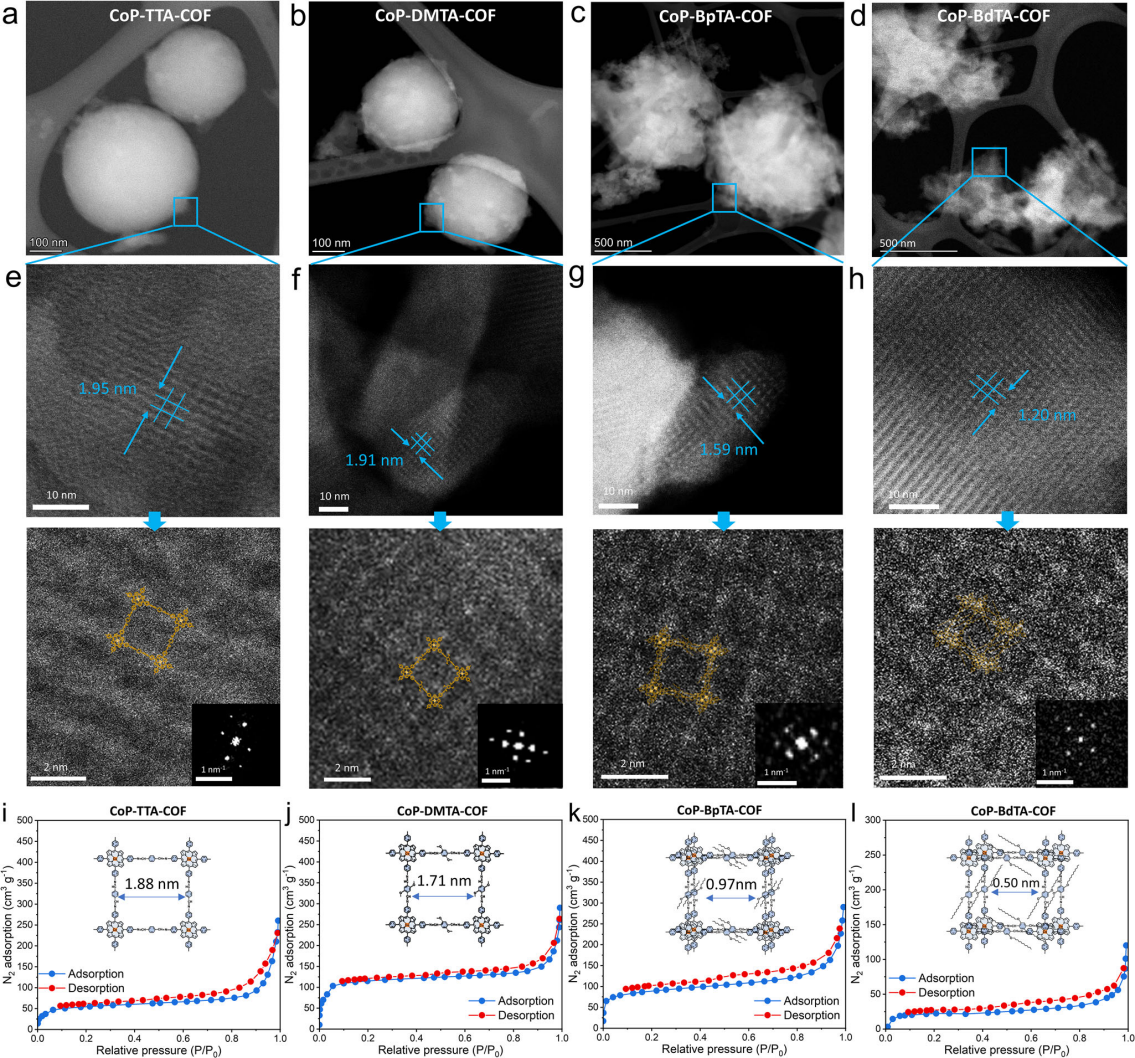
Morphology and porosity evolution of Co‐Por‐COFs. HAADF‐STEM images of (a) CoP‐TTA‐COF, (b) CoP‐DMTA‐COF, (c) CoP‐BpTA‐COF, and (d) CoP‐BdTA‐COF with (e–h) high resolution results. Nitrogen adsorption–desorption isotherms and corresponding pore size distributions of (i) CoP‐TTA‐COF, (j) CoP‐DMTA‐COF, (k) CoP‐BpTA‐COF, and (l) CoP‐BdTA‐COF.

Brunauer–Emmett–Teller (BET) pore size distributions (Figure S20) show that CoP‐TTA‐COF and CoP‐DMTA‐COF exhibit dominant pore sizes centered at 1.88 and 1.71 nm, respectively. In contrast, the pore sizes of CoP‐BpTA‐COF and CoP‐BdTA‐COF decreased to 0.97 and 0.50 nm. This trend is consistent with the HAADF‐STEM observations and reflects the pore contraction accompanying interlayer slipping. As shown in Figure 2i–l, N_2_ adsorption–desorption isotherms display type I behavior for all CoP‐COFs, characteristic of microporosity. The specific surface areas (SSA) of CoP‐TTA‐COF, CoP‐DMTA‐COF, CoP‐BpTA‐COF, and CoP‐BdTA‐COF are 337.5, 421.6, 297.2, and 58.3 m^2^ g^−1^, respectively. Theoretical simulations were further carried out to verify these results. Figure S21 reveals a gradual decrease in both the calculated specific surface area and pore size with increasing side‐chain length across the four COFs. Among these COFs, CoP‐TTA‐COF shows the highest theoretical SSA and a pore size of 1.94 nm. By contrast, CoP‐BdTA‐COF, which contains the longest C_12_ alkoxy chains, exhibits the most severe slip‐stacking, leading to the lowest theoretical surface area and a markedly narrowed pore size of only 0.35 nm. The pore size distributions are generally consistent with the actual BET measurements and follow the same trend, confirming that the increasingly pronounced AA* stacking is induced by the elongation of the side‐chains.

Notably, the experimentally measured SSA of CoP‐DMTA‐COF exceeds that of CoP‐TTA‐COF, deviating from the theoretically predicted monotonic decrease in SSA with increasing alkoxy‐chain length across the CoP‐COFs. This discrepancy can be attributed to the morphological changes induced by the alkoxy side‐chains. SEM images reveal that CoP‐TTA‐COF displays smooth and compact spherical particles, whereas CoP‐DMTA‐COF, after the introduction of the C_1_ side‐chain, maintains a spherical morphology but exhibits a much rougher and more corrugated surface (Figure S18). This roughened surface can expose more pore entrances, leading to the highest experimentally measured SSA. As the alkoxy chains are further extended, the spherical aggregates of CoP‐BpTA‐COF and CoP‐BdTA‐COF become increasingly loose and partially collapsed, resulting in decreased SSA. This phenomenon is especially pronounced for CoP‐BdTA‐COF. Apart from the collapsed morphology, the long C_12_ alkoxy chains can obstruct the pore channels. Together with the slip‐stacking, which narrows the effective pore size to 0.50 nm, N_2_ diffusion into the internal pores is severely restricted. Consequently, both the experimentally measured and theoretically simulated SSA of CoP‐BdTA‐COF are much lower than those of the other three COFs.

### 2.2 Photocatalytic CO_2_RR of CoP‐COFs With Different Side‐Chain Length

All CoP‐COFs were evaluated for photocatalytic CO_2_RR under visible‐light irradiation using [Ru(bpy)_3_]Cl_2_ as the photosensitizer (PS) and triethanolamine (TEOA) as the sacrificial agent (SA). As shown in Figure 3a, both Co‐Por‐NH_2_ and CoP‐COFs can effectively catalyze the photoreduction of CO_2_ to CO. Co‐Por‐NH_2_ exhibits a low CO selectivity of 58.2%, which can be attributed to the amino substituents, which act as Lewis basic sites that facilitate proton adsorption and promote the competing HER. In contrast, all CoP‐COFs show substantially enhanced CO_2_‐to‐CO conversion efficiency and selectivity. Specifically, CO production rates of CoP‐TTA‐COF and CoP‐DMTA‐COF reach 24.5 and 47.5 mmol g^−1^ h^−1^, with selectivity of 74.5% and 80.2%, respectively. Notably, CoP‐BpTA‐COF equipped with a C_5_ side‐chain achieves the highest performance, delivering a CO production rate of 71.4 mmol g^−1^ h^−1^ with a selectivity of 90%. In contrast, CoP‐BdTA‐COF shows a significantly reduced CO formation rate of 19.9 mmol g^−1^ h^−1^, although maintaining the high selectivity of 90.5%. Collectively, these results reveal an optimal alkoxy‐chain‐length effect, with moderate slipped stacking (C_5_) yielding the best performance.

**FIGURE 3:**
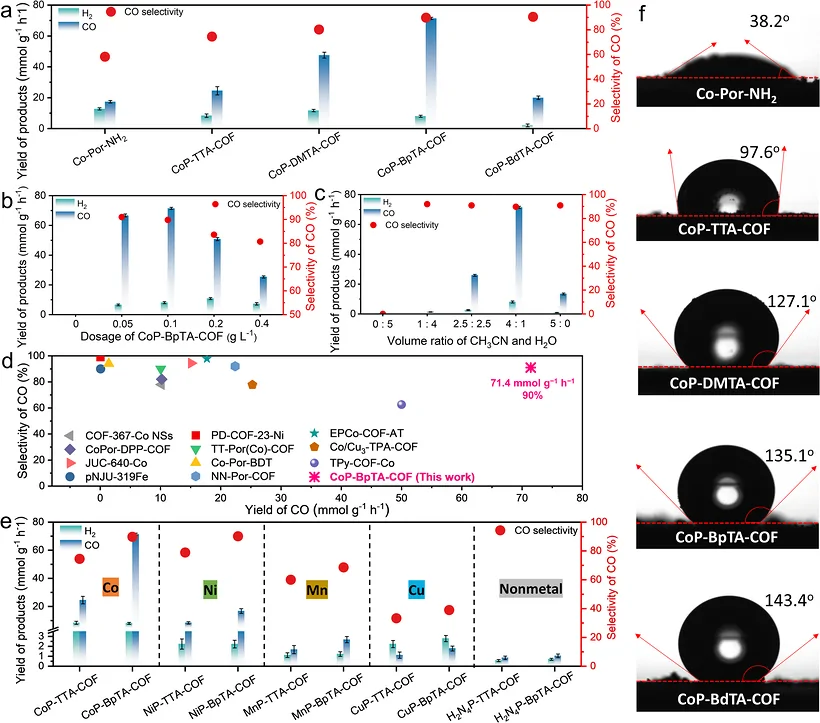
Photocatalytic CO_2_RR and hydrophobicity of CoP‐COFs. (a) CO evolution rates and selectivity over Co‐Por‐NH_2_ and CoP‐COFs. CO evolution rates and selectivity with (b) different dosages of CoP‐BpTA‐COF or (c) different solvent ratios. (d) Comparison of CO evolution rates and selectivity with reported porphyrin‐based COF photocatalysts. (e) CO evolution rates and selectivity over different metalloporphyrin‐COFs in this work. (f) Water contact angles of Co‐Por‐NH_2_ and CoP‐COFs.

A systematic evaluation of the sample stability was performed. First, thermogravimetric analysis (TGA) shows that all COFs remain thermally stable below 400°C under an N_2_ atmosphere, with smaller mass losses than the molecular monomer Co‐Por‐NH_2_ (Figure S22a). We then selected the optimized CoP‐BpTA‐COF for further stability tests, including immersion in strongly acidic (HCl or H_2_SO_4_, pH = 1) and strongly alkaline solutions (NaOH, pH = 12) for 24 h, as well as a long‐term photocatalytic CO_2_RR reaction. As shown in the PXRD patterns in Figure S22b, CoP‐BpTA‐COF retains a certain degree of crystallinity after these different treatments. UV–vis DRS spectra also indicate that its optical properties remain essentially unchanged (Figure S22c). Figure S22d shows that the CO production rate and selectivity of CoP‐BpTA‐COF do not decrease significantly after exposure to these harsh conditions. These results collectively demonstrate that the as‐synthesized CoP‐BpTA‐COF possesses excellent thermal and chemical stability, highlighting its potential for practical application in CO_2_ conversion from flue gas.

Cyclic stability tests were carried out using the optimized catalyst, CoP‐BpTA‐COF. As shown in Figure S23a, the CO production rate and CO selectivity remain nearly unchanged after five consecutive catalytic cycles, indicating good recyclability of CoP‐BpTA‐COF. FTIR spectra, SEM images, PXRD patterns, and UV–vis DRS spectra of CoP‐BpTA‐COF before and after the reaction show no appreciable changes (Figure S23b–e). These results confirm that the chemical structure, morphology, crystallinity, and optical properties of CoP‐BpTA‐COF are well maintained during photocatalytic CO_2_RR, demonstrating its excellent stability and reusability.

The optimized sample, CoP‐BpTA‐COF, was further evaluated under varying photocatalytic CO_2_RR conditions. As shown in Figure 3b,c, the optimal catalyst loading and solvent composition were determined to be 0.1 g L^−1^ and CH_3_CN/H_2_O = 4:1, respectively. Under the optimal conditions, a CO production rate of 71.4 mmol g^−1^ h^−1^ with a selectivity of 90% was achieved, representing a record performance among reported porphyrin‐based COF photocatalysts (Figure 3d and Table S1). Moreover, when the central Co site in the COF was replaced with Ni, Mn, or Cu using the same synthetic protocol, CoP‐COFs consistently delivered the highest activity (Figure 3e). Notably, for the Ni‐, Mn‐, and Cu‐metallated porphyrin COFs, the side‐chain‐modified COFs (MP‐BpTA‐COFs) consistently exhibit superior CO_2_RR‐to‐CO activity and selectivity compared with the side‐chain‐free COFs (MP‐TTA‐COFs). Crystallographic analysis via XRD confirms that C_5_ alkoxy side‐chains induce an interlayer slip in the AA stacking mode (Figure S24), transforming the conventional AA stacking into a serrated AA* stacking configuration. This structural evolution markedly enhances photocatalytic CO_2_RR performance, further demonstrating that side‐chain‐mediated moderate stacking is a highly generalizable strategy for enhancing activity across various metalloporphyrin COF systems. Control experiments demonstrated that no gaseous products were detected in the absence of PS, SA, catalyst, or light irradiation (Figure S25). When CO_2_ was replaced by N_2_, only H_2_ was generated, while CO formation was completely suppressed. Furthermore, the metal‐free H_2_N_4_P‐BpTA‐COF produced negligible amounts of both H_2_ and CO, indicating that the coordinated metal centers act as the active sites. Isotopic labelling experiments analyzed by GC–MS (Figure S26) revealed a distinct ^13^CO signal (*m/z* = 29) when ^12^CO_2_ was replaced with ^13^CO_2_, unambiguously confirming that the CO produced in the system originates from CO_2_.

The enhanced CO_2_RR selectivity observed for CoP‐BpTA‐COF and CoP‐BdTA‐COF is associated with more effective suppression of HER. This behavior originates from the increased hydrophobicity introduced by long alkoxy side‐chains. Indeed, the water contact angle increases from 38.2° for Co‐Por‐NH_2_ to above 90° for all COP‐COFs, confirming a pronounced surface transition from hydrophilic to hydrophobic, which effectively reconstructs the local reaction microenvironment (Figure 3f). In particular, the water contact angles of CoP‐BpTA‐COF and CoP‐BdTA‐COF reach 135.1° and 143.4°, respectively, substantially higher than those of CoP‐TTA‐COF (97.6°) and CoP‐DMTA‐COF (127.1°). These results indicate that the incorporation of C_5_ and C_12_ side‐chains markedly enhances the hydrophobicity of the COF surface and nanochannels, thereby effectively suppressing the adsorption of H_2_O molecules at the catalytic sites.

Collectively, these results demonstrate that alkoxy side‐chain engineering effectively modulates mass transfer process within the COF frameworks, thereby optimizing the photocatalytic CO_2_RR performance even if a decreased BET SSA by introducing alkoxy side‐chains. Notably, CoP‐BpTA‐COF exhibits substantially higher catalytic activity than CoP‐TTA‐COF and CoP‐DMTA‐COF despite its lower SSA (297.2 vs 337.5 and 421.6 m^2^ g^−1^). This discrepancy highlights that side‐chain modification not only influences SSA, but also modulates the degree of AA* stacking slippage. Consequently, this structural evolution critically governs pore channel penetrability and the microenvironment around the central metal sites.

We employed molecular dynamics (MD) simulations to investigate the diffusion behavior of H_2_O and CO_2_ within the CoP‐COFs. Firstly, as shown in Figure 4a, a reduced density of H_2_O molecules near the catalyst surface, with increasing side‐chain length in CoP‐COFs, suppresses the competing HER, which is consistent with water contact angle results. Taking CoP‐BpTA‐COF as an example, Figure 4b illustrates that CO_2_ molecules, initially distributed randomly, gradually accumulate at the COF surface and subsequently diffuse through the micropores into the stacked COF frameworks. Representative snapshots for all four CoP‐COFs at a simulation time of 200 ns are shown in Figure S27. CO_2_ molecules enriched at the surface (within 5 Å of the exterior) are denoted as CO_2_‐surface, while those diffusing into the COF bulk are designated as CO_2_‐bulk. As shown in Figure 4c, CO_2_ diffuses almost unhindered through the non‐slipped pores of CoP‐TTA‐COF and CoP‐DMTA‐COF, leaving less than 10% of molecules at the surface. This indicates that CoP‐COFs with conventional AA stacking affords fully open channels that impose minimal mass‐transfer resistance, enabling highly efficient CO_2_ diffusion. It is noteworthy that C_1_‐functionalized CoP‐DMTA‐COF exhibits a CO_2_ diffusion efficiency nearly identical to that of the unsubstituted CoP‐TTA‐COF (C_0_). This suggests that the alkoxy chains themselves neither chemically adsorb CO_2_ nor sterically impede its diffusion through the COF framework.

**FIGURE 4:**
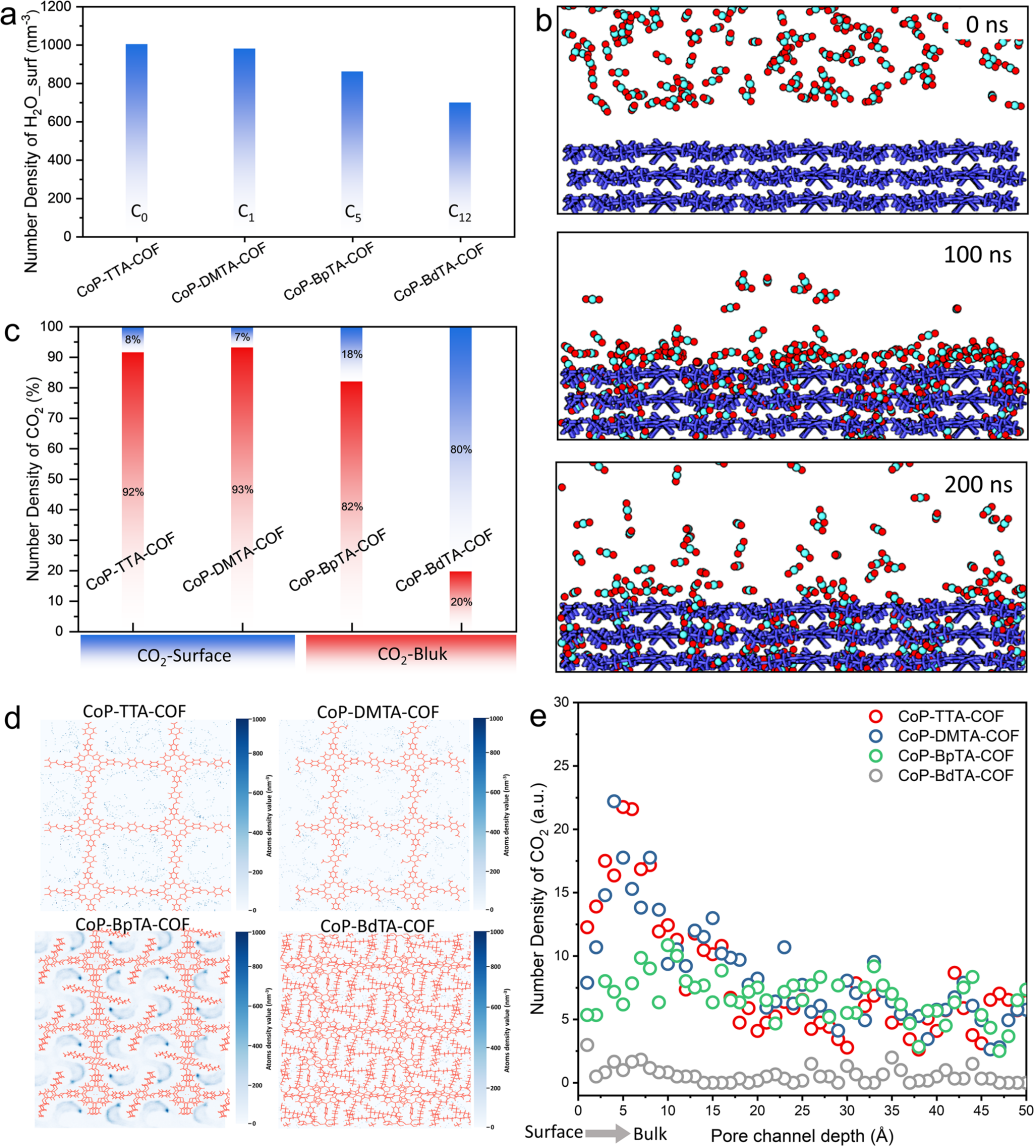
MD simulated H_2_O and CO_2_ distribution in CoP‐COFs. (a) Surface density of H_2_O molecules on CoP‐COFs. (b) Distribution of CO_2_ passing through CoP‐BpTA‐COF at different times (Cyan, red, and blue represent C atoms, O atoms, and CoP‐COFs, respectively, and water molecules are hidden). (c) Number density of CO_2_ accumulated on the surface and diffused in the bulk phase of CoP‐COFs. (d) Heatmap of density distribution for CO_2_ over the CoP‐COFs surface. (e) Density distribution for CO_2_ in the bulk of CoP‐COFs at different pore‐channel depths.

In contrast, for CoP‐BpTA‐COF incorporated with C_5_ side‐chains, the introduced interlayer slippage (AA* stacking) slightly compromises pore connectivity and, consequently, this structure offset mildly restricts CO_2_ diffusion into the bulk framework. As a result, the fraction of CO_2_ retained at the external surface increases to 18%, which is higher than that observed for CoP‐TTA‐COF and CoP‐DMTA‐COF. Nonetheless, because the structural offset in CoP‐BpTA‐COF remains moderate, pore connectivity is largely preserved, allowing the majority of CO_2_ to permeate the COF inner channels. In contrast, CoP‐BdTA‐COF, featuring excessively long C_12_ side‐chains, undergoes a drastic degree of interlayer slippage. Coupled with the steric filling of the longer chains, this severely restricts pore accessibility: only ∼20% of CO_2_ are able to enter the bulk, while the remaining ∼80% accumulate at the external surface. Simulated CO_2_ concentration maps further corroborate these distinct transport behaviors (Figure 4d). In the frameworks with accessible channels (CoP‐TTA‐COF, CoP‐DMTA‐COF, and CoP‐BpTA‐COF), CO_2_ preferentially localizes along the inner pore walls. Notably, CoP‐BpTA‐COF exhibits the highest local CO_2_ population, with CO_2_ adopting a helical distribution around the porphyrin center guided by the side‐chains. In contrast, CO_2_ on the blocked CoP‐BdTA‐COF displays a featureless and uniformly dispersed pattern, directly demonstrating how severe steric restriction of pore connectivity prevents CO_2_ from penetrating into the COF bulk.

It is well established that photocatalytic CO_2_RR relies on the cooperative contribution of active sites located both at the surface and within the bulk of the catalyst. From a perspective of surface catalysis, the slight interlayer slippage in CoP‐BpTA‐COF partially compromises pore continuity, consequently enriching local CO_2_ density at the surface. Compared to CoP‐TTA‐COF and CoP‐DMTA‐COF, the higher surface CO_2_ concentration over CoP‐BpTA‐COF facilitates its adsorption and activation at the exposed Co centers. However, the number of surface Co sites is inherently limited, while the far more abundant Co centers embedded in the bulk ultimately govern the overall CO_2_ uptake and conversion efficiency. Excessive surface accumulation, as observed for CoP‐BdTA‐COF, blocks access to surface Co sites and deprives the bulk of available CO_2_ molecules. This dual detrimental effect accounts for the markedly inferior CO_2_RR activity of CoP‐BdTA‐COF. As shown in Figure 4e, the concentration profiles of CO_2_‐bulk reveal that the bulk CO_2_ levels in CoP‐BdTA‐COF are substantially lower than those in the other three CoP‐COFs with well‐connected channels. Moreover, in CoP‐TTA‐COF and CoP‐DMTA‐COF, CO_2_ primarily accumulates near the subsurface region (approximately 0–10 Å below the surface pore). In striking contrast, CO_2_ is more uniformly distributed throughout the bulk of CoP‐BpTA‐COF, indicating that CO_2_ can penetrate deeper into the nanochannels, driven by the confinement effect of slip‐induced pore contraction. This enhanced infiltration sustains a higher effective adsorption flux across the internal active sites, leading to its superior photocatalytic CO_2_RR performance.

CO_2_ adsorption isotherms of the COFs (Figure S28) further verify the above results. All samples exhibit a gradual increase in CO_2_ uptake as pressure rises, indicating the presence of accessible CO_2_ adsorption sites. Among the four COFs, CoP‐BpTA‐COF shows the highest CO_2_ uptake, reaching 0.13 mmol g^−1^, followed by CoP‐DMTA‐COF, CoP‐TTA‐COF, and CoP‐BdTA‐COF. This trend suggests that the moderately slipped AA* stacking configuration in CoP‐BpTA‐COF may create a confined and hydrophobic pore environment, which favors CO_2_ enrichment. In contrast, CoP‐TTA‐COF and CoP‐DMTA‐COF lack long alkoxy side‐chains and possess relatively large pores, leading to weaker confinement of CO_2_. For CoP‐BdTA‐COF, the overly long alkoxy chains can block the pore channels, while the excessive AA* slipped stacking further narrows the pore aperture, thereby reducing the CO_2_‐accessible pore volume.

### 2.3 Investigation of Photocatalytic Active Sites in CoP‐COFs

Beyond the kinetic advantage arising from confinement‐enhanced CO_2_ diffusion and improved active‐sites utilization, the stacking slip also effectively regulates the electronic features of the catalytic Co sites. The electronic structure and spin state of Co centers in CoP‐COFs were investigated by X‐ray absorption spectroscopy. As shown in Figure 5a, the X‐ray absorption near‐edge structure (XANES) spectra demonstrate that the Co K‐edge absorption energies of the Co‐Por‐NH_2_ and CoP‐COFs closely match that of CoPc, indicating that the Co centers predominantly adopt a divalent oxidation state (Co^2+^). The *k* ^3^‐weighted Fourier‐transformed (FT) extended X‐ray absorption fine structure (EXAFS) spectra in R space (Figure S29) exhibit a pronounced peak at about 1.53 Å, corresponding to the Co─N scattering path, confirming the Co─N coordination environment within the porphyrins. The peak of Co─N path is completely different from the Co─Cl path, suggesting no Co─Cl coordination has formed in the CoP‐COFs and Co‐Por‐NH_2_. A noticeable shift of the Co─N peak toward higher R value is observed for all CoP‐COFs compared with pristine Co‐Por‐NH_2_, with CoP‐BpTA‐COF exhibiting the greatest shift. This positive shift indicates an elongation of the Co─N bond length caused by the weakening of Co─N crystal field strength. Quantitative EXAFS fitting further reveals coordination numbers and bond length of Co─N_4_ for all samples (Figures S30–S34 and Tables S2–S6). Consistently, the inset of Figure 5a shows that the pre‐edge features of the CoP‐COFs are significantly broadened compared to Co‐Por‐NH_2_, indicating that the Co 3*d* levels are split into several closely spaced unoccupied states. Such splitting implies a weakened crystal‐field strength in CoP‐COFs compared with the monomeric Co porphyrin. Given that the oxidation state and coordination environment of the Co centers remain unchanged, this effect is most likely attributed to an increased spin state of Co^2+^ [39, 40].

**FIGURE 5:**
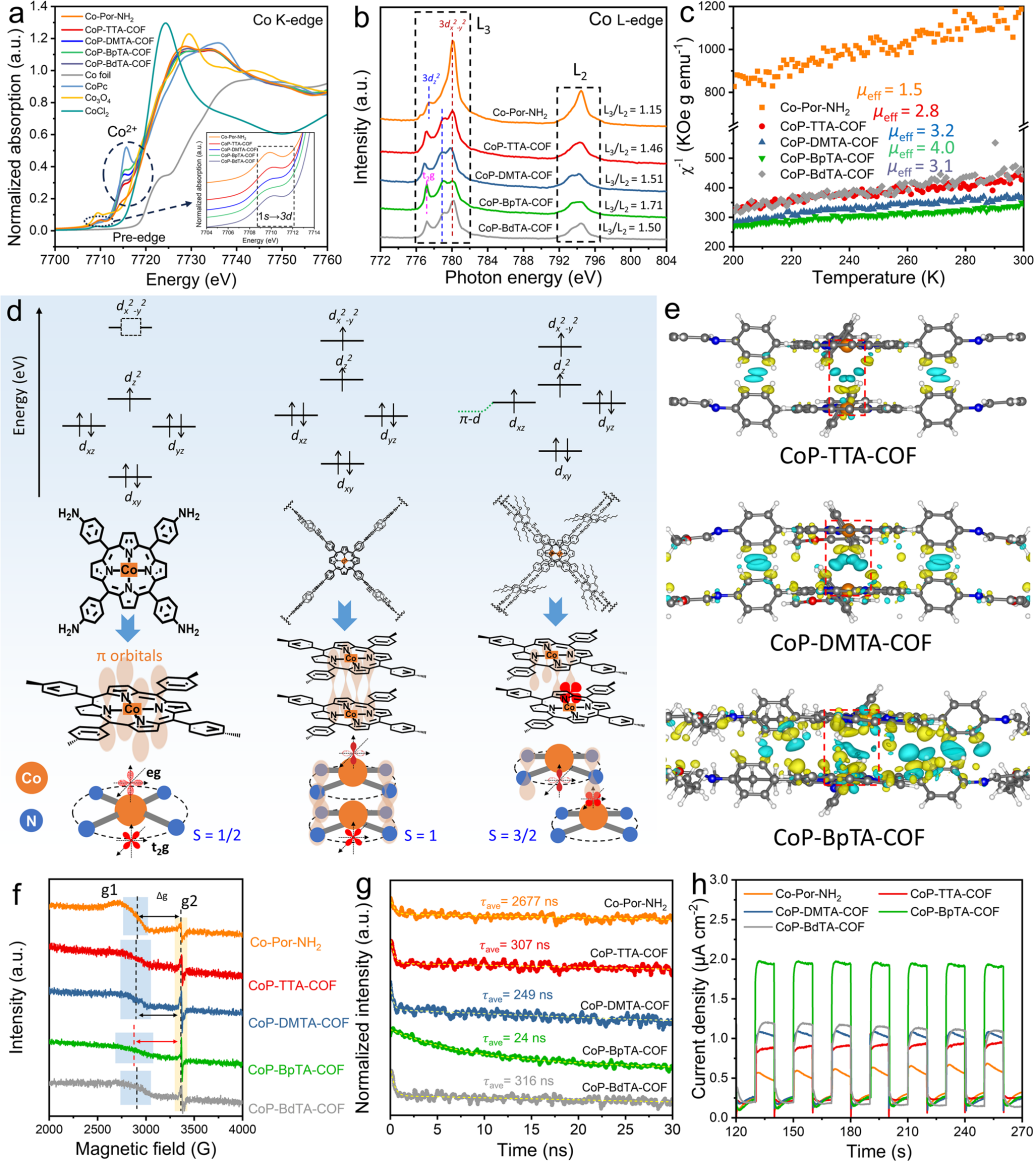
Electronic structure and spin‐state modulation of CoP‐COFs. (a) XANES and (b) NEXAFS spectra of Co‐Por‐NH_2_ and CoP‐COFs. (c) Curie−Weiss fitting of M–T curves for Co‐Por‐NH_2_ and CoP‐COFs. (d) 3*d* orbital diagrams of Co‐Por‐NH_2_ and CoP‐COFs with AA stacking and AA* stacking. (e) Charge density distributions of CoP‐COFs (Yellow and cyan parts represent the increased charge density and decreased charge density, respectively). (f) Solid EPR of Co‐Por‐NH_2_ and CoP‐COFs. (g) Fluorescence lifetimes of PS in the presence of Co‐Por‐NH_2_ and CoP‐COFs. (h) Transient photocurrent responses of Co‐Por‐NH_2_ and CoP‐COFs.

The near‐edge XAFS (NEXAFS) spectra in Figure 5b further show that all samples exhibit two characteristic features at the Co L‐edge: an L_3_ edge at approximately 780 eV, corresponding to the transition from Co 2*p_3/2_* to unoccupied 3*d* orbital, and an L_2_ edge at approximately 794 eV, corresponding to the transition from Co 2*p_1/2_* to 3*d* [41, 42]. The occupancy of the Co 3*d* orbitals and the resulting multiple structure, both dictated by the Co spin state, are sensitively reflected in the L‐edge spectral features. In the case of monomeric Co‐Por‐NH_2_, the L_3_‐edge spectrum is dominated by the transition from Co 2*p_3/2_* to the 3*d_x_^2^‐_y_^2^* orbital, with only a minor contribution from the 2*p_3/2_* to 3*d_z_^2^* transition. The predominance of 3*d_x_^2^_‐y_^2^*‐related peak indicates that this orbital is essentially unoccupied due to a high energy, implying that the Co^2+^ center in Co‐Por‐NH_2_ adopts a low‐spin configuration. By contrast, the line shape of L_2,3_‐edges changes markedly for all CoP‐COFs: the L_2,3_‐edge shifts toward lower binding energies, and a prominent low‐energy shoulder emerges, associated with the transition from 2*p_3/2_* to t_2_g (3*d_xz_*, 3*d_yz_*, and 3*d_xy_*). This spectral evolution indicates that, in the COF frameworks, a larger fraction of spin electrons occupies 3*d_x_^2^_‐y_^2^* orbitals, which have lower energy than that of Co‐Por‐NH_2_, signifying an overall increase in the Co^2+^ spin state. Notably, since the 2*p* core hole interacts with 3*d* electrons through exchange coupling when unpaired 3*d* electrons are present, an enhanced L_3_/L_2_ ratio can be attributed to the increased number of unpaired electrons, reflected by an elevation of spin state [43]. Quantitative analysis of L_3_/L_2_ area ratios (Figure 5b) verifies that the spin state of Co in the CoP‐COFs is higher than that in Co‐Por‐NH_2_, with CoP‐BpTA‐COF exhibiting the highest spin‐state population at Co^2+^ centers. Consistent with these observations, the Co 2*p* core‐level X‐ray photoelectron spectra (XPS) reveal significantly enhanced 2*p_3/2_* satellite features for CoP‐COFs compared to Co‐Por‐NH_2_ (Figure S35), further confirming the higher density of unpaired electrons within the COF‐coordinated Co sites.

The spin and magnetic properties of metal centers were quantitatively evaluated by temperature‐dependent magnetic susceptibility (M–T) measurements. In M–T curves, the magnetic susceptibility decreases monotonously with increased temperatures, implying a typical paramagnetic behavior over all samples (Figure S36). As shown in Figure 5c, the inverse susceptibility (χ^−1^) shows a linear dependence on temperature, indicative of Curie–Weiss behavior according to Equation 1. H denotes the externally applied magnetic field, fixed at 0.1 T, and M represents the magnetic susceptibility, reported in units of emu g^−1^. Curie–Weiss law defines the paramagnetic component using Equation 2, where C is Curie constant and *θ*_Weiss_ is the paramagnetic Curie temperature (the Weiss constant). The effective paramagnetic moment, *µ*_eff_, is obtained from the Curie–Weiss fit using the Equation 3, where *m* is molar mass of samples [44, 45]. The obtained *µ*_eff_ value directly reflects the spin state of the metal ions according to Equation 4 (Lande factor g = 2.0 for 3*d* transition metals, *S* is spin quantum number) [46, 47].

(1) $$\chi^{-1}=H/M$$

(2) $$\chi =C/(T-\theta_{Weiss})$$

(3) $$\mu_{eff}=2.828\sqrt{mC}\mu_{B}$$

(4) $$\mu_{eff}=g\sqrt{S\left(S+1\right)}\mu_{B}$$

Based on the calculated *µ*_eff_ by Figure 5c, the corresponding *S* of each sample can be quantitatively assigned (Table 1).

**TABLE 1:** Quantitative spin state of each sample.

Samples	*µ*_eff_	*S*	Spin state
Co‐Por‐NH_2_	1.5	1/2	Low spin
CoP‐TTA‐COF	2.8	1	Intermediate spin
CoP‐DMTA‐COF	3.2	1	Intermediate spin
CoP‐BpTA‐COF	4.0	3/2	High spin
CoP‐BdTA‐COF	3.1	1	Intermediate spin

The quantitatively extracted spin states are fully consistent with the NEXAFS analysis. Co^2+^ center in Co‐Por‐NH_2_ adopts a low‐spin configuration (*S* = 1/2). Upon incorporation of porphyrin monomer into COF lattice, Co^2+^ sites experience a pronounced spin‐state elevation. Notably, CoP‐BpTA‐COF exhibits the highest spin state (*S* = 3/2), whereas the rest of the functionalized CoP‐COFs display intermediate‐spin configurations (*S* = 1). This spin‐state evolution originates from variations in the coordination field induced by different structural configurations. As shown in Figure 5d, the central Co in the monomer Co‐Por‐NH_2_ follows a rigid N_4_ coordination environment with a very strong in‐plane conjugated crystal field. The energy splitting between the *d_x_^2^_‐y_^2^* and *d_z_^2^* orbitals is large, leading Co^2+^ to adopt a low‐spin state. At this stage, due to the absence of interlayer magnetic exchange, the Co^2+^ sites exhibit a completely isolated single‐ion spin state. After the formation of the ordered framework, however, the π–π conjugation interactions in the AA‐stacked structure weaken the in‐plane Co─N crystal field strength, lowering the energy level of *d_x_^2^_‐y_^2^* and raising the spin state to intermediate spin. When the AA stacking of COF undergoes moderate slipping (CoP‐BpTA‐COF), the structural symmetry is disrupted, and the Co─Co interaction along the vertical direction maximizes ferromagnetic coupling through π–d exchange, further increasing the collective spin state to *S* = 3/2.

Charge differential density analysis further supports this mechanism (Figure 5e). In AA stacked CoP‐TTA‐COF and CoP‐DMTA‐COF, interlayer charge transfer is mainly concentrated on the Co centers. In contrast, CoP‐BpTA‐COF (C_5_) with a moderately slipped structure exhibits much stronger charge transfer at both the Co sites and the porphyrin rings, confirming the vertical interaction between the Co *d* orbitals and the porphyrin π orbitals across the layers. However, excessive slipping in CoP‐BdTA‐COF (C_12_) weakens the π–d exchange pathways between the Co sites and the porphyrin rings, reverting the overall spin state to an intermediate level (Figure S37). Consistently, solid‐state EPR spectra show that CoP‐BpTA‐COF exhibits the broadest low‐field g_1_ feature along the interlayer direction and the largest Δ*g* between low‐field (*g*_1_) and in‐plane high‐field (*g*_2_) signals (Figure 5f). These characteristics collectively reveal that CoP‐BpTA‐COF exhibits the strongest interlayer Co–π–Co exchange interaction among the series. Projected density of states (PDOS) calculated by DFT for CoP‐COFs elucidate that CoP‐BpTA‐COF exhibits the most pronounced splitting of t_2_g orbitals, the highest density of states near the Fermi level, and a clear spin‐asymmetric distribution (Figure S38). This confirms that its π–d perpendicular exchange increases the number of unpaired electrons in the t_2_g orbitals.

To further probe the origin of the performance differences among the CoP‐COFs in photocatalytic CO_2_RR, we investigated their optical properties. As shown in UV–vis DRS (Figure S39a), all COF samples exhibit stronger visible‐light absorption than the Co‐Por‐NH_2_ monomer. On the basis of Tauc plots (Figure S39b), the calculated optical band gap energy (*E*_g_) for Co‐Por‐NH_2_ is 1.6 eV, which is narrower than that of CoP‐COFs (1.75/1.81 eV). Band structures were then probed by Mott−Schottky measurements (Figure S39c), in which the positive slopes indicate that these samples are n‐type semiconductors [48]. The lowest conduction band (CB) positions of all samples are more negative than the theoretical potentials for CO_2_‐to‐CO (−0.52 V vs NHE, pH = 7) and HER (−0.41 V vs NHE, pH = 7), indicating their thermodynamic feasibility for CO_2_RR to CO (Figure S39d).

The high‐spin Co centers typically introduce spin‐forbidden recombination pathways in semiconductors, thereby prolonging carrier lifetimes and facilitating more efficient separation and transport of photogenerated charges. Accordingly, photoluminescence (PL) quenching experiments of PS were conducted to probe charge dynamics occurring during the photocatalytic process. As shown in Figure S40, upon excitation at 465 nm, the PS solution exhibits an emission peak at 625 nm, corresponding to radiative relaxation from the excited state to ground state. PL intensity is markedly quenched upon the introduction of catalysts. Among all catalysts, CoP‐BpTA‐COF, which features the highest spin state at the Co centers, exhibits the most pronounced PL quenching. This observation indicates its superior ability to intercept, and transfer photoexcited electrons from PS, thereby effectively suppressing relaxation of PS back to its ground state.

To investigate the electron transfer kinetics, various catalyst dosages were employed to evaluate their PL‐quenching effects on PS (Figure S41). The calculated quenching constants increase systematically with the spin state of Co centers, highlighting that higher‐spin Co sites more effectively extract and utilize photoexcited electrons to mitigate charge recombination. Furthermore, the addition of TEOA effectively functions as an electron SA [49, 50]. Based on these findings, the overall photocatalytic process is proposed in Figure S42. Under light irradiation, PS is excited to its active PS* state, which subsequently injects electrons into catalysts, generating a PS^+^ intermediate. Catalysts then transfer and utilize these electrons to drive catalytic reaction. Meanwhile, SA supplies electrons to regenerate ground‐state PS, thereby sustaining the continuous catalytic turnover.

To quantitatively probe the dynamics of this interfacial electron transfer, time‐resolved fluorescence lifetimes of PS were measured in the presence of different catalysts. As shown in Figure 5g, all CoP‐COFs accelerate the fluorescence decay of PS, compared to the isolated Co‐Por‐NH_2_ monomer. Notably, CoP‐BpTA‐COF yields the most pronounced lifetime quenching; this rapid depopulation of the PS excitonic state is attributed to the high‐spin Co centers, which act as formidable electron sinks. By facilitating ultrafast interfacial electron extraction through non‐radiative pathways, these high‐spin sites effectively suppress bulk recombination, thereby prolonging the lifetime of separated charge carriers for catalytic charge utilization [51]. The transient photocurrent responses (Figure 5h) and electrochemical impedance spectra (Figure S43) further corroborate this spin‐dependent kinetic advantage, confirming that the collective high‐spin state in CoP‐BpTA‐COF exhibits the most efficient photogenerated charge separation and transport. This phenomenon extends to other transition‐metal‐based porphyrin COFs as well. In the NEXAFS spectra, all MP‐BpTA‐COFs show an increased L_3_/L_2_ branching ratio compared with their corresponding MP‐TTA‐COFs, suggesting modification of the metal 3d electronic configuration (Figure S44a–c). For Ni‐ and Mn‐coordinated samples, the increased L_3_/L_2_ ratio can be interpreted as an increase in the spin state of the metal centers [52, 53]. For Cu‐coordinated samples, the increased L_3_/L_2_ reflects enhanced Cu 3*d*‐hole character and modifications in the Cu─N ligand‐field environment [54, 55]. These electronic‐structure changes can promote the transfer and utilization of photogenerated electrons at the metal sites (Figure S44d–f).

For CO_2_RR, Co sites in CoP‐COFs with serrated slip‐stacking structures can optimize both CO_2_ adsorption and the formation of reaction intermediates. As illustrated in Figure 6a, DFT calculations identify *COOH formation as the rate‐determining intermediate along the CO_2_‐to‐CO pathway. Among the CoP‐COFs, CoP‐BpTA‐COF exhibits the lowest reaction barrier for *COOH formation. In situ FTIR spectroscopy reveals the formation of bidentate carbonate (b‐CO_3_^2−^, 1260 cm^−1^) and monodentate carbonate (m‐CO_3_^2−^, 1430 cm^−1^) [56, 57]. Meanwhile, a dominant *COOH feature at 1123 and 1476 cm^−1^ emerges and intensifies progressively, serving as a pivotal intermediate toward CO formation (Figure 6b) [58, 59]. In addition, Co‐Por‐NH_2_ and all CoP‐COFs exhibit the identical suite of detectable reaction intermediates, indicating a conserved catalytic pathway across all samples (Figure S45). After normalizing the *COOH peak evolution, it becomes evident that CoP‐BpTA‐COF exhibits the fastest formation rate of *COOH. This kinetic acceleration is facilitated by the highest collective spin state of Co sites, consistent with its superior CO_2_‐to‐CO conversion efficiency (Figure S46).

**FIGURE 6:**
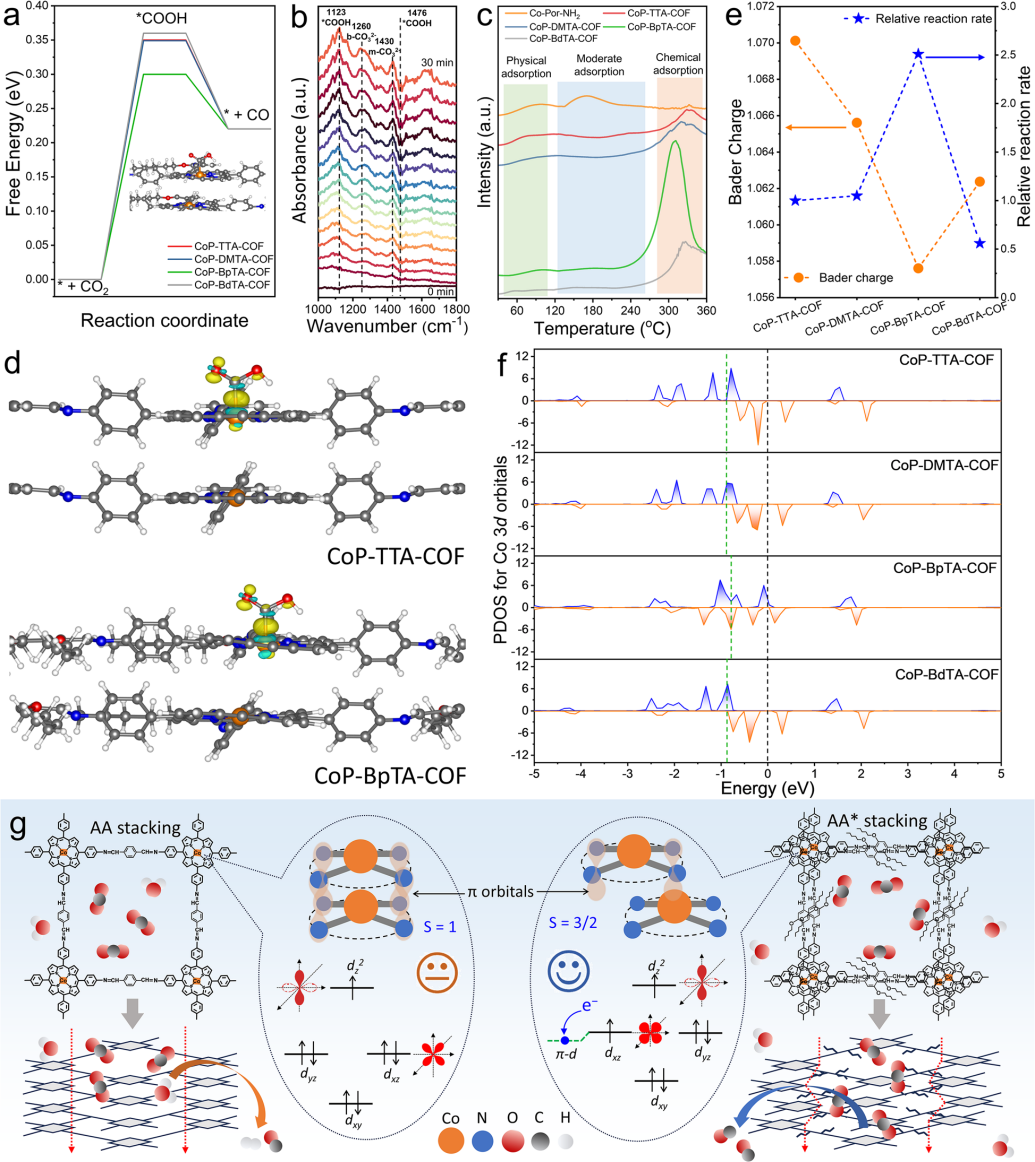
CO_2_ adsorption and activation by CoP‐COFs. (a) Free energy diagram of CoP‐COFs for CO_2_RR. (b) In situ FTIR spectra of CoP‐BpTA‐COF. (c) CO_2_‐TPD of Co‐Por‐NH_2_ and CoP‐COFs. (d) Charge density distributions of CoP‐TTA‐COF and CoP‐BpTA‐COF with *COOH (Yellow and cyan parts represent the increased charge density and decreased charge density, respectively). (e) Bader charges and relative reaction rates of Co‐Por‐NH_2_ and CoP‐COFs. (f) PDOS of CoP‐COFs. (g) Mechanism diagram of alkoxy side‐chain‐mediated stacking regulation over CoP‐COFs for confined CO_2_ diffusion and high‐spin effect.

Figure 6c presents the temperature‐programmed desorption profiles for CO_2_ (CO_2_‐TPD), revealing that low‐spin Co‐Por‐NH_2_ exhibits predominantly physisorption and moderate chemisorption toward CO_2_. In contrast, all CoP‐COFs with medium‐ to high‐spin Co centers display pronounced chemisorption. Among them, CoP‐BpTA‐COF shows the strongest chemisorption capacity, attributable to the elevated *d*‐orbital electron density of high‐spin Co sites, which enhances their Lewis basicity and intensifies the interaction with CO_2_, forming a stable Co─CO_2_ adduct and promoting electron donation to activate the CO_2_ molecule. The differential charge density maps further confirm that upon *COOH adsorption, Co sites in CoP‐BpTA‐COF accumulate and redistribute charge more effectively than those in CoP‐TTA‐COF (Figure 6d). Moreover, the lowest Bader charge of CoP‐BpTA‐COF further indicates that its Co sites possess the highest electron density, enabling the fastest CO_2_RR reaction kinetics (Figure 6e). In Figure 6f, the green dashed line represents the position of d‐band center relative to the Fermi level. Based on the *d*‐band theory [60], the d‐band center of CoP‐BpTA‐COF is closest to the Fermi level (Figure S47), indicating the strongest adsorption of intermediates over CoP‐BpTA‐COF.

Moreover, to rule out the direct influence of the C_5_ alkoxy chain itself on the Co sites, we synthesized a noncrystalline porous organic polymer (CoP‐BpTA‐POP). XRD confirms its fully amorphous nature without ordered interlayer registry (Figure S48). NEXAFS and M–T curve fitting further reveal that Co centers in CoP‐BpTA‐POP retain the same low‐spin state (*S* = 1/2) as the monomer Co‐Por‐NH_2_ (Figures S49 and S50). Consequently, its photocatalytic CO_2_RR activity is comparable to that of Co‐Por‐NH_2_ and dramatically inferior to that of CoP‐BpTA‐COF (Figure S51). In addition, PDOS and free‐energy calculations comparing the slipped and eclipsed configurations of CoP‐BpTA‐based materials corroborate that the side‐chain–induced interlayer slip, rather than the mere presence of the alkoxy chains, intrinsically accounts for the enhanced photocatalytic CO_2_RR performance through spin‐state enhancement (Figures S52 and S53).

Based on these findings, a unified mechanism for enhanced photocatalytic CO_2_RR in side‐chain‐engineered COFs is proposed in Figure 6g. To accommodate the steric bulk of long alkoxy chains (≥C_5_), the typically eclipsed AA stacking of porphyrin COFs undergoes a serrated slip (AA* stacking) to achieve thermodynamically structural stabilization. This structural evolution unlocks two kinetic advantages. First, the moderate slip induced by the medium‐length C_5_ side‐chain finely tunes the pore penetrability; this not only increases the local CO_2_ concentration at the external surface for rapid activation but also promotes more homogeneous CO_2_ diffusion throughout the COF framework through a confinement effect. In addition, the nonpolar, long alkoxy chains inherently enhance the local hydrophobicity of COF skeleton, suppressing competitive HER and thereby securing superior CO_2_RR selectivity. From an electronic perspective, transforming Co‐Por‐NH_2_ monomer into an ordered COF framework weakens in‐plane Co─N ligand field through strong π–π stacking, initially raising Co centers to an intermediate spin state. Once the framework adopts the side‐chain‐induced serrated AA* stacking, the resulting reduction in symmetry triggers a strong π–d‐mediated Co─Co magnetic exchange. This interaction maximizes ferromagnetic coupling, further elevating the COF to a collective high‐spin state. Crucially, this high‐spin effect serves a dual function: it facilitates superior interlayer charge transfer by extending the lifetime of active charge carriers, and it energetically enhances CO_2_ adsorption and activation at the metal centers. In summary, the AA* stacking engineered via alkoxy side‐chains over CoP‐COFs boosts photocatalytic CO_2_RR across three synergistic dimensions: increasing surface site utilization, enabling uniform confined CO_2_ diffusion, and activating a high‐spin catalytic center to overcome activation barriers.

## 3 Conclusion

In conclusion, we present a side‐chain‐directed interlayer serrated sliding strategy to synchronously engineer the catalytic microenvironment and electronic spin configuration in CoP‐COFs for highly efficient photocatalytic CO_2_RR‐to‐CO. By incorporating sterically demanding alkoxy substituents, the conventional eclipsed AA stacking is transformed into a precisely controlled slipped configuration, generating profound structural, and electronic consequences. The moderately serrated sliding simultaneously optimizes pore continuity and penetrability while enhancing hydrophobic confinement, thereby enriching local CO_2_ concentration and actively suppressing the competing HER. More fundamentally, the interlayer registry reconstruction modulates the Co ligand field and reinforces vertical π–d exchange interactions, driving a collective spin‐state transition from the low‐spin monomer to a more active high‐spin framework state. The moderately slipped stacking structures promote spin‐dependent charge separation and prolong carrier lifetimes, ultimately accelerating CO_2_ adsorption, activation, and reduction kinetics. Beyond performance improvement, this work establishes serrated interlayer sliding as a versatile structural engineering strategy to integrate spin‐state regulation with catalytic microenvironment engineering for rational design of next‐generation organic framework photocatalysts for solar‐to‐chemical energy conversion.

## Author Contributions

**Jie He**: conceptualization, data curation, investigation, formal analysis, Writing – original draft, validation. **Qingxuan Chen**: investigation, and methodology. **Minxian Zhang**: investigation and formal analysis. **Wenhao Zhao**: data curation. **Aoni Xu**: methodology, investigation, and software. **Jinqiang Zhang**: conceptualization, formal analysis, supervision, and writing – review & editing. **Hongqi Sun**: formal analysis. **Shaobin Wang**: formal analysis, supervision, and writing – review & editing. **Xiaoguang Duan**: writing – review & editing, conceptualization, funding acquisition, resources, supervision, and formal analysis.

## Funding

This work is funded by the Australian Research Council via Discovery scheme (DE250100753, FT230100526, and DP230102406).

## Conflicts of Interest

The authors declare no conflicts of interest.

## Supporting information




**Supporting File**: adma74747‐sup‐0001‐SuppMat.docx.

## Data Availability

The data that support the findings of this study are available from the corresponding author upon reasonable request.
