# Supplementary material for: Side‐Chain‐Induced Interlayer Slipping in Metalloporphyrin COFs Enables Microenvironment and Spin‐State Regulation for Photocatalytic CO2 Reduction
Source: Adv Mater. 2026 Aug 22;38(58):e74747. doi: 10.1002/adma.74747 (PMC13674631; doi:10.1002/adma.74747)
Supplement: Supplementary file 1 [file ADMA-38-e74747-s001.docx]

**Supporting Information for**

Side-Chain-Induced Interlayer Slipping in Metalloporphyrin COFs Enables Microenvironment and Spin-State Regulation for Photocatalytic CO_2_ Reduction

Jie He,^1^ Qingxuan Chen,^2^ Minxian Zhang,^1^ Wenhao Zhao,^3^ Aoni Xu,^2^* Jinqiang Zhang,^3^* Hongqi Sun,^3^ Shaobin Wang,^1^ Xiaoguang Duan,^1^*

^1^ School of Chemical Engineering, Adelaide University, Adelaide, SA 5005, Australia

^2^ School of Chemical and Biomolecular Engineering and ARC Centre of Excellence for Green Electrochemical Transformation of Carbon Dioxide, The University of Sydney, Sydney, NSW, 2006 Australia

^3^ School of Molecular Sciences, The University of Western Australia, Perth, WA 6009, Australia

* Correspondence and requests for materials should be addressed to: A.N. Xu (aoni.xu@sydney.edu.au), J.Q. Zhang ([jinqiang.zhang@uwa.edu.au](mailto:jinqiang.zhang@uwa.edu.au)), X.G. Duan ([xiaoguang.duan@adelaide.edu.au](mailto:xiaoguang.duan@adelaide.edu.au)).

**Supporting Text**

**Text 1. Chemicals.** Cobalt chloride hexahydrate (CoCl_2_⋅6H_2_O, 99%), 5,10,15,20-Tetrakis-(4-aminophenyl)-porphyrin (H_2_N_4_-Por-NH_2_),  terephthalaldehyde (99%), 2,5-dimethoxybenzene-1,4-dicarboxaldehyde (97%), 2,5-bis(pentyloxy)terephthalaldehyde (98%), 2,5-bis(dodecyloxy)terephthalaldehyde (98%), sodium sulfite (Na_2_SO_4_, 99.8%), methanol (99.8%), ethanol (99.8%), acetone (99.8%), tetrahydrofuran (THF, 99%), *N,N*-dimethylformamide (DMF, 99%), 1,2-dichlorobenzene (o-DCB, 99%), n-butanol (98%), acetic acid (98%), tris(2,2'-bipyridyl) dichlororuthenium(II) hexahydrate ([Ru(bpy)_3_]Cl_2_·6H_2_O), acetonitrile (CH_3_CN, 99%), and triethanolamine (TEOA, 98%) were purchased from Sigma Aldrich platform. All chemicals were of analytical grades and used as received without further purification.

**Text 2. Characterizations.** Morphology and element distributions of the samples were observed by Scanning Electron Microscope (SEM, Hitachi SU7000). Crystal structure of powder product was investigated *via* X-ray diffraction (PXRD, Rigaku MiniFlex 600) D2 PHASER with Cu-Kα radiation with 2-theta degree from 2 to 60. Optical absorption behaviors were measured by UV-vis spectrometer (Cary Series, Agilent Technologies). Thermo Scientific K-Alpha X-ray photoelectron spectroscopy system (Thermo Fisher Scientific, UK) was used to detect XPS signals. HAADF STEM imaging and EDX mapping were captured on a FEI Titan Themis 80-200 (200 kV voltage) equipped with a Super-X high-sensitivity windowless EDX detector. Solid-state ^13^C CP/MAS NMR spectrum were recorded on a Bruker Avance NEO 400 MHZ NMR spectrometer. Fourier-transform infrared (FTIR) spectra were obtained using a Nicolet iS50 spectrometer from ThermoFisher. Photoluminescence (PL) spectra were measured on a FLS1000 spectrometer. Water contact angles were measured using an Attension Theta optical tensiometer. Solid electron paramagnetic resonance (EPR) spectra were recorded on a Magnettech ESR5000 spectrometer. X-ray absorption data were collected on the X-ray absorption spectroscopy (XAS) and soft X-ray spectroscopy (SXRS) beamline at Australian Synchrotron (Melbourne). All X-ray absorption spectra were measured in a transition mode at room temperature, and the data were processed and analyzed using the QANT software program and ATHENA/ARTEMIS module of IFEFFIT software package.

**Text 3. Electrochemical process.** Transient photocurrents responses and electrochemical impedance spectra (EIS) were carried out on a three-electrode cell (CHI 760E, Shanghai Chenhua) using a standard three-electrode configuration. Working electrode was prepared by drop-casting a dispersion of catalyst (2 g L^−1^ in water-enthanol solution) containing 10 μL Nafion onto a glassy carbon electrode. An Ag/AgCl electrode and a Pt wire were used as reference and counter electrodes, respectively. A 0.2 mol L^−1^ Na_2_SO_4_ solution (pH=7) was used as electrolyte.

Text 4. Synthesis of Co-Por-NH_2_. Co-Por-NH_2_ was synthesized according to a modified literature procedure [1-2]. Specifically, 404.88 mg of 5,10,15,20-Tetrakis-(4-aminophenyl)-porphyrin (H_2_N_4_-Por-NH_2_) was dispersed in 35 mL of *N,N*-dimethylformamide (DMF) (denoted as solution A). CoCl_2_⋅6H_2_O (178.45 mg) was then added, and the mixture was stirred under a N_2_ atmosphere at 50 °C for 30 min. The reaction temperature was subsequently raised to 150 °C and maintained under reflux for 3 h. After cooling to room temperature, 50 mL of ultrapure water was added to precipitate the product. The solid was collected by suction filtration, washed three times with ultrapure water and methanol, and dried under vacuum at 60 °C for 12 h.

Text 5. Synthesis of CoP-COFs. CoP-COFs were synthesized via a typical solvothermal Schiff base polycondensation. The preparation of CoP-BpTA-COF is described as a representative example. 5,10,15,20-Tetrakis(4-aminophenyl) porphyrin-cobalt(II) (Co-Por-NH_2_, 36.6 mg, 0.05 mmol), 2,5-bis(pentyloxy)terephthalaldehyde (30.6 mg, 0.10 mmol), 1,2-dichlorobenzene (1 mL), and n-butanol (1 mL) were added to a heat-resistant glass tube equipped with a Schlenk system. The mixture was ultrasonicated for 10 min to afford a homogeneous suspension, followed by the addition of acetic acid (0.2 mL, 6 M). After another 5 min of ultrasonication to ensure complete dissolution of the precursors, the tube was subjected to three freeze–pump–thaw cycles to remove dissolved gases. The tube was then sealed under vacuum and heated at 120 °C for 72 h in an oven. After cooling to room temperature, the precipitated solid was collected by vacuum filtration and washed thoroughly with acetone and tetrahydrofuran (THF) under ultrasonication. The crude product was further purified by Soxhlet extraction with THF for 24 h and finally dried under vacuum to yield the CoP-BpTA-COF as a dark purple powder.

CoP-TTA-COF, CoP-DMTA-COF and CoP-BdTA-COF were synthesized using the same procedure, except that terephthalaldehyde, 2,5-dimethoxybenzene-1,4-dicarboxaldehyde and 2,5-bis(dodecyloxy)terephthalaldehyde were used, respectively, in place of 2,5-bis(pentyloxy)terephthalaldehyde.

Text 6. Synthesis of CoP-BpTA-POP. Co-Por-NH_2_ (14.6 mg, 0.02 mmol) and 2,5-bis(pentyloxy)terephthalaldehyde (17.9 mg, 0.04 mmol) were dissolved in o-DCB (1 mL) in a Pyrex tube (10 mL). The mixture was degassed via three freeze-pump-thaw cycles, sealed under vacuum, and heated at 120 °C for 72 h. The resulting precipitate was collected by filtration, washed with THF, and further purified by Soxhlet extraction with THF at 85 °C for 24 h. The dark purple powder was collected and dried at room temperature under vacuum overnight to afford CoP-BpTA-POP (yield: 85%).

**Text 7. Evaluation of photocatalytic CO_2_RR performance.** Photocatalytic CO_2_RR was conducted in a 125 mL sealed Pyrex reactor equipped with a quartz window. Typically, 0.5 mg of catalyst was dispersed in a mixed solvent of acetonitrile (CH_3_CN) and H_2_O (v/v, 4:1, total 5 mL), containing [Ru(bpy)_3_]Cl_2_ (7 mg) as photosensitizer (PS) and triethanolamine (TEOA, 0.5 mL) as sacrificial agent. The reactor was sealed and purged with high-purity CO_2_ (99.999%) for 20 min to ensure complete saturation (0.05 MPa). The reactor was irradiated with a 300 W Xe lamp (CEL-PF300-T8, Au-light, Beijing) equipped with a 420 nm-cut filter, at a light intensity of 200 mW cm^−2^. A circulating water system was employed to maintain the reaction temperature at 25±1 ^o^C. The gaseous products were analyzed using a gas chromatograph (Agilent 8890 GC System) equipped with a flame ionization detector (FID) and a thermal conductivity detector (TCD). Isotope labelling experiments were performed under identical conditions using ^13^CO_2_ instead of CO_2_. The selectivity toward CO_2_ reduction (SCO_2_) was calculated as follows: [3]

SCO_2_ = NCO_2_RR /(NCO_2_RR + N_HER_)*100%

**Text 8. In-situ FTIR experiments.** In-situ FTIR measurements were performed on a Nicolet iS50 FTIR spectrometer (Thermo Fisher, USA) equipped with a variable angle specular reflection accessory (VeeMAX III, PIKE) and a custom-designed photocatalytic reaction cell with quartz windows. Typically, 1 mg of catalyst powder was dispersed in 1 mL of ethanol and ultrasonicated to form a uniform suspension. The suspension was drop-cast onto a silicon wafer and dried to form a thin catalyst film. The silicon substrate was then mounted in the *in situ* cell, followed by the addition of 1 mL of reaction solution (CH_3_CN/H_2_O, containing photosensitizer and TEOA). The cell was purged with CO_2_ for 30 min to remove residual air. A MCT/A detector was used, and background spectra were collected until the CO_2_ peak at 2370 cm^−1^ stablilized. A spectrum under dark conditions (0 min) was recorded as the baseline. Then, the Xenon lamp equipped with an optical fiber was aligned with the quartz window of the reaction cell, and the light source was turned on to initiate the reaction, during which the infrared spectra within the wavenumber range of 400-4000 cm^−1^ was collected at set time intervals.

**Text 9. Computational method**

DFT calculations were performed with Vienna Ab Initio Simulation Package (VASP) [4]. Revised Perdew-Burke-Ernzerhof (PBE) functional [5] and dispersion correction using the DFT-D3 method with the Becke-Johnson damping function [6]. The Inner electrons were represented by projector-augmented wave method [7]. A plane-wave basis set was adopted with an energy cut-off of 520 eV for valence electrons. Only one k-point is employed for the axis along the normal direction of the surface. The computational hydrogen electrode model was used to calculate the energy of H^+^ and OH^−^ ions [8].

The reaction pathways for CO are summarized as Eq. S1-3.

CO_2_ + * → *CO_2_	Eq. S1

*CO_2_ + H^+^ + e^−^ → *COOH	Eq. S2

*COOH + H^+^ + e^−^ → *CO + H_2_O	Eq. S3

The relative reaction rate (k) is calculated using the Arrhenius equation (Eq. S4). In this equation, k is the reaction rate, A is the pre-exponential factor, E is the energy barrier of the reaction, R is the molar gas constant (8.314 J/(mol·K)), and T is the absolute temperature.

k = Ae^−E/RT^	Eq. S4

Molecular dynamics (MD) were carried out to study the distribution of H_2_O and CO_2_ on the material surface. The visualization was performed using Visual Molecular Dynamics (VMD) software [9]. All MD simulations were performed using GROMACS (version 2025.2) [10]. The system was modeled with a hybrid force field approach. All elements except cobalt use the general Amber Force Field (GAFF) [11], while cobalt uses the Universal Force Field (UFF) forcefield.[12] Water molecules were represented explicitly using the TIP3P model [13]. CO_2_ is described by the TRAPPE_small Force field [14]. The energy minimization procedure was run until maximum force was below 1000 kJ/mol on every atom with steepest descent integrator [15]. Temperature was maintained at 303.15 K using the v-rescale thermostat with the canonical ensemble (NVT) [16]. In order to constrain hydrogen bonds, the LINCS constraint algorithm was used [17]. Short-range nonbonded interactions were truncated at 1.2 nm, with a Verlet cutoff scheme [18]. Van der Waals forces were smoothed using a force-switch modifier between 1.0 nm and 1.2 nm. Long-range electrostatics were treated with Particle Mesh Ewald (PME) [19]. In the molecular dynamics simulation system, the material surface consists of six layers of a 3×3 structure on both the top and bottom. In the four systems, the number of CO_2_ molecules is 600 in each, while the number of water molecules is as follows: 20,992 (CoP-TTA-COF); 20,914 (CoP-DMTA-COF); 20,604 (CoP-BpTA-COF); and 19,202 (CoP-BdTA-COF). Each system undergoes a 200 ns simulation after energy minimization, and all data are averaged over the top and bottom surfaces. Molecules within 5 Å of the surface are adsorbed near the surface, while molecules below the surface are present in the bulk phase.

**Supporting Figures**

**Figure S1.** Diagram of synthetic procedure for CoP-COFs with different side-chain lengths.

**Figure S2.** Solid-state^13^C CP/MAS NMR spectrum and peak assignment of Co-Por-NH_2_.

**Figure S3.** Solid-state^13^C CP/MAS NMR spectrum and peak assignment of CoP-TTA-COF.

**Figure S4.** Solid-state^13^C CP/MAS NMR spectrum and peak assignment of CoP-DMTA-COF.

**Figure S5.** Solid-state^13^C CP/MAS NMR spectrum and peak assignment of CoP-BpTA-COF.

**Figure S6.** Solid-state^13^C CP/MAS NMR spectrum and peak assignment of CoP-BdTA-COF.

**Figure S7.** FTIR spectra with ATR mode for porphyrin and COFs samples.

**Figure S8.** Experimental PXRD patterns of monomeric precursors.

**Figure S9.** Experimental PXRD patterns of Co-Por-NH_2_.

**Figure S10.** (a) PXRD patterns of CoP-TTA-COF compared with different stacking modes. Reproduced structures of CoP-TTA-COF using (b) AA stacking with α = 90° and (c) AA* stacking with 1.5 Å slippage.

**Figure S11.** PXRD patterns of CoP-DMTA-COF compared with AA* stacking mode with 1.5 Å slippage.

**Figure S12.** (a) PXRD of CoP-COFs, (b) enlarged figure of (a). (c) High-resolution PXRD of CoP-COFs, (d) enlarged figure of (c).

**Figure S13.** PXRD patterns of CoP-BpTA-COF compared with different stacking modes and their corresponding reproduced structures.

**Figure S14.** PXRD patterns of CoP-BdTA-COF compared with different stacking modes and their corresponding reproduced structures.

**Figure S15.** PXRD patterns of (a) CoP-TTA-COF, (b) CoP-DMTA-COF, (c) CoP-BpTA-COF and (d) CoP-BdTA-COF with experimental curves, Pawley refinement profiles, corresponding difference plots, simulated patterns for AA stacking, AA* stacking with different slippage distance and AB stacking mode, and Bragg reflection positions.

**Figure S16.** (a) Relative energies of CoP-COFs with non-slipped AA-stacking structures and slipped AA* stacking structures. Energy of (b) CoP-TTA-COF and (c) CoP-BpTA-COF with different interlayer slip distances.

**Figure S17.** PXRD, Pawley refinement and Rietveld refinement with refined structures of (a) CoP-BpTA-COF and (b) CoP-BdTA-COF.

**Figure S18.** SEM images of (a) CoP-TTA-COF, (b) CoP-DMTA-COF, (c) CoP-BpTA-COF, (d) CoP-BdTA-COF.

**Figure S19.** HAADF-STEM image of Co-Por-NH_2_.

**Figure S20.** Pore size distributions of CoP-COFs.

**Figure S21.** Theoretical SSA and pore size distributions of CoP-COFs.

**Figure S22.** (a) Thermal gravimetric analysis (TGA) curves of samples. (b) PXRD, (c) UV–vis DRS and (d) CO_2_RR performance of CoP-BpTA-COF with different treatments.

**Figure S23.** (a) Cyclic tests of photocatalytic CO_2_RR by CoP-BpTA-COF. (b) FTIR spectra, (c) SEM images, (d) PXRD patterns, and (e) UV–vis DRS spectra of CoP-BpTA-COF before and after reaction.

**Figure S24.** PXRD patterns of (a) NiP-TTA-COF, NiP-BpTA-COF, (b) MnP-TTA-COF, MnP-BpTA-COF, (c) CuP-TTA-COF and CuP-BpTA-COF with corresponding difference plots.

**Figure S25.** Control experiments of photocatalytic CO_2_RR.

**Figure S26.** Isotope experiments of photocatalytic CO_2_RR tested by GC-MS.

**Figure S27.** MD simulated CO_2_ distribution over CoP-COFs.

**Figure S28.** CO_2_ adsorption isotherms of (a) CoP-TTA-COF, (b) CoP-DMTA-COF, (c) CoP-BpTA-COF and (d) CoP-BdTA-COF.

**Figure S29.** EXAFS spectra of Co-Por-NH_2_, CoP-COFs and standard Co samples in R space.

**Figure S30.** EXAFS fitting curves of Co-Por-NH_2_ in R-space and k-space.

**Figure S31.** EXAFS fitting curves of CoP-TTA-COF in R-space and k-space.

**Figure S32.** EXAFS fitting curves of CoP-DMTA-COF in R-space and k-space.

**Figure S33.** EXAFS fitting curves of CoP-BpTA-COF in R-space and k-space.

**Figure S34.** EXAFS fitting curves of CoP-BdTA-COF in R-space and k-space.

**Figure S35.** XPS spectra in Co 2p core level.

**Figure S36.** M-T curves over Co-Por-NH_2_ and CoP-COFs.

**Figure S37.** Intemediate spin diagram by excessive AA* stacking and the charge density distribution of CoP-BdTA-COF.

**Figure S38.** PDOS of eg and t_2_g for 3*d* orbitals of CoP-COFs.

**Figure S39.** (a) UV–vis DRS spectra, (b) Tauc plots, (c) Mott-Schottky plots and (d) energy band structures of samples.

**Figure S40.** PL quenching effect of PS by different samples.

**Figure S41.** PL quenching effect of PS by (a) Co-Por-NH_2_, (b) CoP-TTA-COF, (c) CoP-DMTA-COF, (d) CoP-BpTA-COF and (e) CoP-BdTA-COF with different catalyst dosage. (f) Quenching constants of different catalysts.

**Figure S42.** Quencing diagram of PS in photocatalytic condition with catalysts.

**Figure S43.** Electrochemical impedance spectroscopy of samples.

**Figure S44.** L-edge NEXAFS of (a) Ni-, (b) Mn-, and (c) Cu-coordinated porphyrin COFs. Transient photocurrent responses of (d) Ni-, (e) Mn-, and (f) Cu-coordinated porphyrin COFs.

**Figure S45.** In situ FTIR spectra of Co-Por-NH_2_ and CoP-COFs.

**Figure S46.** In situ FTIR signal intensity of *COOH spectra over Co-Por-NH_2_ and CoP-COFs.

**Figure S47.** D band center of CoP-COFs.

**Figure S48.** XRD pattern of CoP-BpTA-POP.

**Figure S49.** NEXAFS spectra of Co L-edge among Co-Por-NH_2_, CoP-BpTA-POP and CoP-BpTA-COF.

**Figure S50.** Comparison of temperature-dependent magnetic susceptibility with the Curie−Weiss fittings among Co-Por-NH_2_, CoP-BpTA-POP and CoP-BpTA-COF.

**Figure S51.** Comparison of CO yield and selectivity among Co-Por-NH_2_, CoP-BpTA-POP and CoP-BpTA-COF in photocatalytic CO_2_RR.

**Figure S52.** PDOS of eg and t_2_g for 3*d* orbitals of CoP-BpTA-COF with AA* or AA stacking structures.

**Figure S53.** Free energy comparision fo CO_2_RR for CoP-BpTA-COF with AA* and AA stacking.

**Supporting Tables**

**Table S1.** Porphyrin- or COFs-based photocatalysts for CO_2_RR. AA (ascorbic acid); TIPA (triisopropanolamine); BIH (1,3-dimethyl-2-phenyl-2,3-dihydro-1H-benzo[d]imidazole; TEOA (triethanolamine); TEA (trimethylamine).

| **Samples** | **Porphyrins** | **Reaction**  **medium** | **Photosensitizer** | **Sacrificial**  **agent** | **CO**  **selectivity** | **Yield rate of CO**  **(**μmol g^−1^ h^−1^**)** | **Ref** |
| --- | --- | --- | --- | --- | --- | --- | --- |
| COF-367-Co NSs | CoPor | 0.1 M KHCO_3_ solution | [Ru(bpy)_3_]Cl_2_ | AA | 78% | 10162 | [20] |
| CoPor-DPP-COF | CoPor | CH_3_CN/H_2_O | [Ru(bpy)_3_]Cl_2_ | TIPA | 82% | 10200 | [21] |
| JUC-640-Co | CoPor | CH_3_CN/H_2_O | [Ru(bpy)_3_]Cl_2_ | BIH | 94.4% | 15100 | [22] |
| pNJU-319Fe | FePor | CH_3_CN/H_2_O | / | TEOA | 90% | 68.8 | [23] |
| PD-COF-23-Ni | NiPor | CH_3_CN/H_2_O | / | TEOA | 99% | 40 | [24] |
| TT-Por(Co)-COF | CoPor | CH_3_CN/H_2_O | [Ru(bpy)_3_]Cl_2_ | TEOA | 90% | 10050 | [25] |
| Co-Por-BDT | CoPor | CH_3_CN | Re-(bpy)(CO)_3_Cl | TEOA | 94% | 1424 | [26] |
| NN-Por-COF | CoPor | CH_3_CN/H_2_O | [Ru(bpy)_3_]Cl_2_ | TIPA | 92% | 22380 | [27] |
| EPCo-COF-AT | CoPc | CH_3_CN/H_2_O | [Ru(bpy)_3_]Cl_2_ | TEOA | 97.8% | 17700 | [28] |
| Co/Cu_3_-TPA-COF | / | CH_3_CN/H_2_O | [Ru(bpy)_3_]Cl_2_ | TEOA | 77.8% | 25248 | [29] |
| TPy-COF-Co | / | CH_3_CN/H_2_O | [Ru(bpy)_3_]Cl_2_ | TEOA | 62.6% | 50000 | [30] |
| CoP-BpTA-COF | CoPor | CH_3_CN/H_2_O | [Ru(bpy)_3_]Cl_2_ | TEOA | 90% | 71428 | This work |

**Table S2.** EXAFS curve fitting parameters for Co K-edge of Co-Por-NH_2_.

| Path | d^b^/ Å (Reff) | R / Å | *N* | σ^2^ / Å^2^ |
| --- | --- | --- | --- | --- |
| Co-N | 1.953 | 1.941 ± 0.01 | 4 | 0.004 |
| Co-C | 2.983 | 2.929 ± 0.01 | 0.7 | 0.010 |
| Co-N-C | 3.146 | 3.244 ± 0.02 | 0.5 | 0.027 |

**Table S3.** EXAFS curve fitting parameters for Co K-edge of CoP-TTA-COF.

| Path | d^b^/ Å (Reff) | R / Å | *N* | σ^2^ / Å^2^ |
| --- | --- | --- | --- | --- |
| Co-N | 1.953 | 1.951 ± 0.01 | 4 | 0.004 |
| Co-C | 2.983 | 2.973 ± 0.01 | 8 | 0.005 |
| Co-N-C | 3.146 | 3.182 ± 0.02 | 28 | 0.028 |

**Table S4.** EXAFS curve fitting parameters for Co K-edge of CoP-DMTA-COF.

| Path | d^b^/ Å (Reff) | R / Å | *N* | σ^2^ / Å^2^ |
| --- | --- | --- | --- | --- |
| Co-N | 1.953 | 1.953 ± 0.01 | 4 | 0.003 |
| Co-C | 2.983 | 2.980 ± 0.01 | 9 | 0.005 |
| Co-N-C | 3.146 | 3.179 ± 0.02 | 26 | 0.021 |

**Table S5.** EXAFS curve fitting parameters for Co K-edge of CoP-BpTA-COF.

| Path | d^b^/ Å (Reff) | R / Å | *N* | σ^2^ / Å^2^ |
| --- | --- | --- | --- | --- |
| Co-N | 1.953 | 1.968 ± 0.01 | 4 | 0.003 |
| Co-C | 2.983 | 2.983 ± 0.01 | 12 | 0.007 |
| Co-N-C | 3.146 | 3.186 ± 0.01 | 36 | 0.024 |

**Table S6.** EXAFS curve fitting parameters for Co K-edge of CoP-BdTA-COF.

| Path | d^b^/ Å (Reff) | R / Å | *N* | σ^2^ / Å^2^ |
| --- | --- | --- | --- | --- |
| Co-N | 1.953 | 1.958 ± 0.01 | 4 | 0.003 |
| Co-C | 2.983 | 2.983 ± 0.01 | 10 | 0.006 |
| Co-N-C | 3.146 | 3.194 ± 0.01 | 32 | 0.025 |

**Supporting References**

1.	M. Yuasa, K. Oyaizu, A. Yamaguchi, et al., “Micellar cobaltporphyrin nanorods in alcohols,” *Journal of the American Chemical Society* 126 (2004): 11128-11129, <https://doi.org/10.1021/ja0486216>

2.	Y.-N. Gong, W. Zhong, Y. Li, et al., “Regulating photocatalysis by spin-state manipulation of cobalt in covalent organic frameworks,” *Journal of the American Chemical Society* 142 (2020): 16723-16731, <https://doi.org/10.1021/jacs.0c07206>

3.	J. Fu, K. Jiang, X. Qiu, et al., “Product selectivity of photocatalytic CO_2_ reduction reactions,” *Materials Today* 32 (2020): 222-243, <https://doi.org/10.1016/j.mattod.2019.06.009>

4.	G. Kresse, J. Furthmüller, “Efficiency of ab-initio total energy calculations for metals and semiconductors using a plane-wave basis set,” *Computational materials science* 6 (1996): 15-50, <https://doi.org/10.1016/0927-0256(96)00008-0>

5.	B. Hammer, L. B. Hansen, J. K. Nørskov, “Improved adsorption energetics within density-functional theory using revised Perdew-Burke-Ernzerhof functionals,” *Physical Review B* 59 (1999): 7413, <https://doi.org/10.1103/PhysRevB.59.7413>

6.	S. Grimme, J. Antony, S. Ehrlich, et al., “A consistent and accurate ab initio parametrization of density functional dispersion correction (DFT-D) for the 94 elements H-Pu,” *The Journal of chemical physics* 132 (2010) <https://doi.org/10.1063/1.3382344>

7.	G. Kresse, D. Joubert, “From ultrasoft pseudopotentials to the projector augmented-wave method,” *Physical Review B* 59 (1999): 1758, <https://doi.org/10.1103/PhysRevB.59.1758>

8.	J. K. Nørskov, J. Rossmeisl, A. Logadottir, et al., “Origin of the overpotential for oxygen reduction at a fuel-cell cathode,” *The Journal of Physical Chemistry B* 108 (2004): 17886-17892, <https://doi.org/10.1021/jp047349j>

9.	W. Humphrey, A. Dalke, K. Schulten, “VMD: visual molecular dynamics,” *Journal of molecular graphics* 14 (1996): 33-38, <https://doi.org/10.1016/0263-7855(96)00018-5>

10.	D. Van Der Spoel, E. Lindahl, B. Hess, et al., “GROMACS: fast, flexible, and free,” *Journal of computational chemistry* 26 (2005): 1701-1718, <https://doi.org/10.1002/jcc.20291>

11.	J. Wang, R. M. Wolf, J. W. Caldwell, et al., “Development and testing of a general amber force field,” *Journal of computational chemistry* 25 (2004): 1157-1174, <https://doi.org/10.1002/jcc.20035>

12.	A. K. Rappé, C. J. Casewit, K. S. Colwell, et al., “UFF, a full periodic table force field for molecular mechanics and molecular dynamics simulations,” *Journal of the American Chemical Society* 114 (1992): 10024-10035, <https://doi.org/10.1021/ja00051a040>

13.	W. L. Jorgensen, J. Chandrasekhar, J. D. Madura, et al., “Comparison of simple potential functions for simulating liquid water,” *The Journal of chemical physics* 79 (1983): 926-935, <https://doi.org/10.1063/1.445869>

14.	B. L. Eggimann, Y. Sun, R. F. DeJaco, et al., “Assessing the quality of molecular simulations for vapor–liquid equilibria: An analysis of the TraPPE database,” *Journal of Chemical* 65 (2019): 1330-1344, <https://doi.org/10.1021/acs.jced.9b00756>

15.	M. J. Abraham, T. Murtola, R. Schulz, et al., “GROMACS: High performance molecular simulations through multi-level parallelism from laptops to supercomputers,” *SoftwareX* 1 (2015): 19-25, <https://doi.org/10.1016/j.softx.2015.06.001>

16.	G. Bussi, D. Donadio, M. Parrinello, “Canonical sampling through velocity rescaling,” *The Journal of chemical physics* 126 (2007) <https://doi.org/10.1063/1.2408420>

17.	B. Hess, H. Bekker, H. J. Berendsen, et al., “LINCS: A linear constraint solver for molecular simulations,” *Journal of computational chemistry* 18 (1997): 1463-1472, <https://doi.org/10.1002/(SICI)1096-987X(199709)18:12><1463::AID-JCC4>3.0.CO;2-H

18.	S. Páll, B. Hess, “A flexible algorithm for calculating pair interactions on SIMD architectures,” *Computer Physics Communications* 184 (2013): 2641-2650, <https://doi.org/10.1016/j.cpc.2013.06.003>

19.	C. Kutzner, S. Páll, M. Fechner, et al., “More bang for your buck: Improved use of GPU nodes for GROMACS 2018,” *Journal of computational chemistry* 40 (2019): 2418-2431, <https://doi.org/10.1002/jcc.26011>

20.	W. Liu, X. Li, C. Wang, et al., “A scalable general synthetic approach toward ultrathin imine-linked two-dimensional covalent organic framework nanosheets for photocatalytic CO_2_ reduction,” *Journal of the American Chemical Society* 141 (2019): 17431-17440, <https://doi.org/10.1021/jacs.9b09502>

21.	X. Wang, X. Ding, T. Wang, et al., “Two-dimensional porphyrin-based covalent organic framework with enlarged inter-layer spacing for tunable photocatalytic CO_2_ reduction,” *ACS applied materials interfaces* 14 (2022): 41122-41130, <https://doi.org/10.1021/acsami.2c12542>

22.	J. Ding, X. Guan, J. Lv, et al., “Three-dimensional covalent organic frameworks with ultra-large pores for highly efficient photocatalysis,” *Journal of the American Chemical Society* 145 (2023): 3248-3254, <https://doi.org/10.1021/jacs.2c13817>

23.	P. Dong, X. Xu, R. Luo, et al., “Postsynthetic annulation of three-dimensional covalent organic frameworks for boosting CO_2_ photoreduction,” *Journal of the American Chemical Society* 145 (2023): 15473-15481, <https://doi.org/10.1021/jacs.3c03897>

24.	N. Xu, Y. Diao, X. Qin, et al., “Donor–acceptor covalent organic frameworks of nickel (ii) porphyrin for selective and efficient CO_2_ reduction into CO,” *Dalton Transactions* 49 (2020): 15587-15591, <https://doi.org/10.1039/D0DT03205K>

25.	S. S. Zhao, J. Liang, D.-H. Si, et al., “Superheterojunction covalent organic frameworks: Supramolecular synergetic charge transfer for highly efficient photocatalytic CO_2_ reduction,” *Applied Catalysis B: Environment Energy* 333 (2023): 122782, <https://doi.org/10.1016/j.apcatb.2023.122782>

26.	Y. H. Kim, J. P. Jeon, Y. Kim, et al., “Cobalt‐porphyrin‐based covalent organic frameworks with donor‐acceptor units as photocatalysts for carbon dioxide reduction,” *Angewandte Chemie* 135 (2023): e202307991, <https://doi.org/10.1002/ange.202307991>

27.	X. Ma, J. Hu, S. Li, et al., “Porphyrin-based covalent organic frameworks with undulated layers for efficient photocatalytic CO_2_ reduction,” *Science Bulletin* 70 (2025): 2277-2284, <https://doi.org/10.1016/j.scib.2025.04.002>

28.	W. Lin, F. Lin, J. Lin, et al., “Efficient photocatalytic CO_2_ reduction in ellagic acid–based covalent organic frameworks,” *Journal of the American Chemical Society* 146 (2024): 16229-16236, <https://doi.org/10.1021/jacs.4c04185>

29.	X. Lan, H. Li, Y. Liu, et al., “Covalent organic framework with donor1‐acceptor‐donor2 motifs regulating local charge of intercalated single cobalt sites for photocatalytic CO_2_ reduction to syngas,” *Angewandte Chemie* 136 (2024): e202407092, <https://doi.org/10.1002/ange.202407092>

30.	P. Fu, C. Chen, C. Wu, et al., “Covalent organic framework stabilized single CoN_4_Cl_2_ site boosts photocatalytic CO_2_ reduction into tunable syngas,” *Angewandte Chemie International Edition* 64 (2025): e202415202, <https://doi.org/10.1002/anie.202415202>
